# SARS-CoV-2 gene content and COVID-19 mutation impact by comparing 44 *Sarbecovirus* genomes

**DOI:** 10.1038/s41467-021-22905-7

**Published:** 2021-05-11

**Authors:** Irwin Jungreis, Rachel Sealfon, Manolis Kellis

**Affiliations:** 1 MIT Computer Science and Artificial Intelligence Laboratory, Cambridge, MA USA; 2 Broad Institute of MIT and Harvard, Cambridge, MA USA; 3 Center for Computational Biology, Flatiron Institute, Simons Foundation, New York, NY USA

**Keywords:** Sequence annotation, Molecular evolution, Comparative genomics, SARS-CoV-2

## Abstract

Despite its clinical importance, the SARS-CoV-2 gene set remains unresolved, hindering dissection of COVID-19 biology. We use comparative genomics to provide a high-confidence protein-coding gene set, characterize evolutionary constraint, and prioritize functional mutations. We select 44 *Sarbecovirus* genomes at ideally-suited evolutionary distances, and quantify protein-coding evolutionary signatures and overlapping constraint. We find strong protein-coding signatures for ORFs 3a, 6, 7a, 7b, 8, 9b, and a novel alternate-frame gene, ORF3c, whereas ORFs 2b, 3d/3d-2, 3b, 9c, and 10 lack protein-coding signatures or convincing experimental evidence of protein-coding function. Furthermore, we show no other conserved protein-coding genes remain to be discovered. Mutation analysis suggests ORF8 contributes to within-individual fitness but not person-to-person transmission. Cross-strain and within-strain evolutionary pressures agree, except for fewer-than-expected within-strain mutations in nsp3 and S1, and more-than-expected in nucleocapsid, which shows a cluster of mutations in a predicted B-cell epitope, suggesting immune-avoidance selection. Evolutionary histories of residues disrupted by spike-protein substitutions D614G, N501Y, E484K, and K417N/T provide clues about their biology, and we catalog likely-functional co-inherited mutations. Previously reported RNA-modification sites show no enrichment for conservation. Here we report a high-confidence gene set and evolutionary-history annotations providing valuable resources and insights on SARS-CoV-2 biology, mutations, and evolution.

## Abstract (web summary)

The SARS-CoV-2 gene set remains unresolved, hindering dissection of COVID-19 biology. Comparing 44 *Sarbecovirus* genomes provides a high-confidence protein-coding gene set. The study characterizes protein-level and nucleotide-level evolutionary constraints, and prioritizes functional mutations from the ongoing COVID-19 pandemic.

## Introduction

**Fig. 1:**
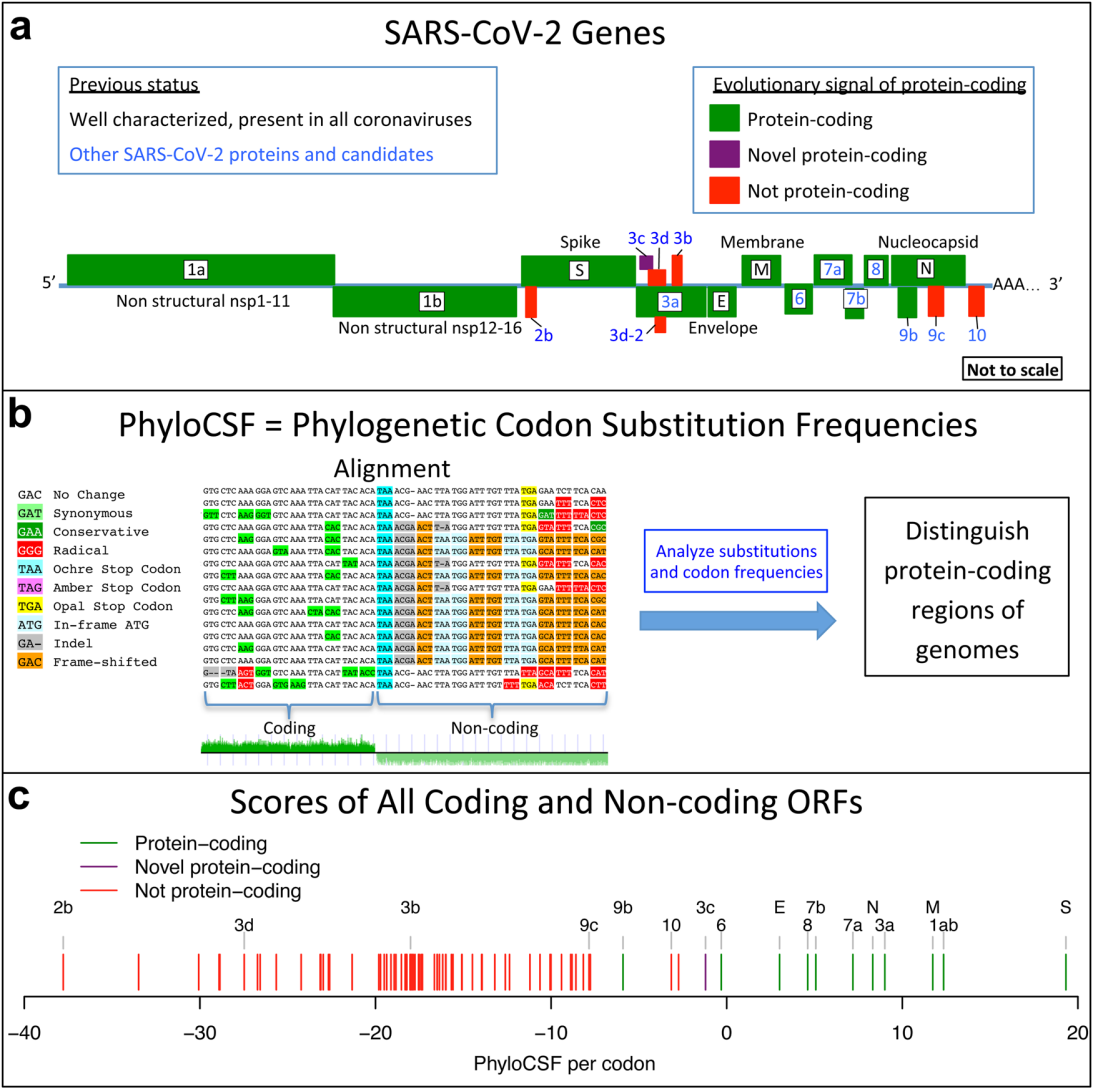
Overview. **a** Coronavirus-wide (black font) and species-specific or candidate (blue font) SARS-CoV-2 genes, with confirmed protein-coding (green), rejected (red), or novel protein-coding (purple) classification, using evolutionary and experimental evidence. **b** Phylogenetic Codon Substitution Frequencies (PhyloCSF) scores distinguish protein-coding (left) vs. non-coding (right) using evolutionary signatures, including distinct frequencies of amino-acid-preserving (green) vs. amino-acid-disruptive (red) substitutions, and stop codons (cyan/magenta/yellow) in frame-specific alignments, and additional features. **c** PhyloCSF score (*x*-axis) for all confirmed (green) and rejected (red) ORFs, showing annotated/candidate/novel (labeled) and all AUG-initiated ≥25-codons-long locally maximal ORFs (unlabeled). Novel ORF3c (purple) clusters with protein-coding. Only modestly negative ORF9c/ORF10 scores are artifacts of score compression in high-nucleotide-constraint regions, and substantially drop when nucleotide-conservation-scaled (see Supplementary Fig. 3).

**Fig. 2:**
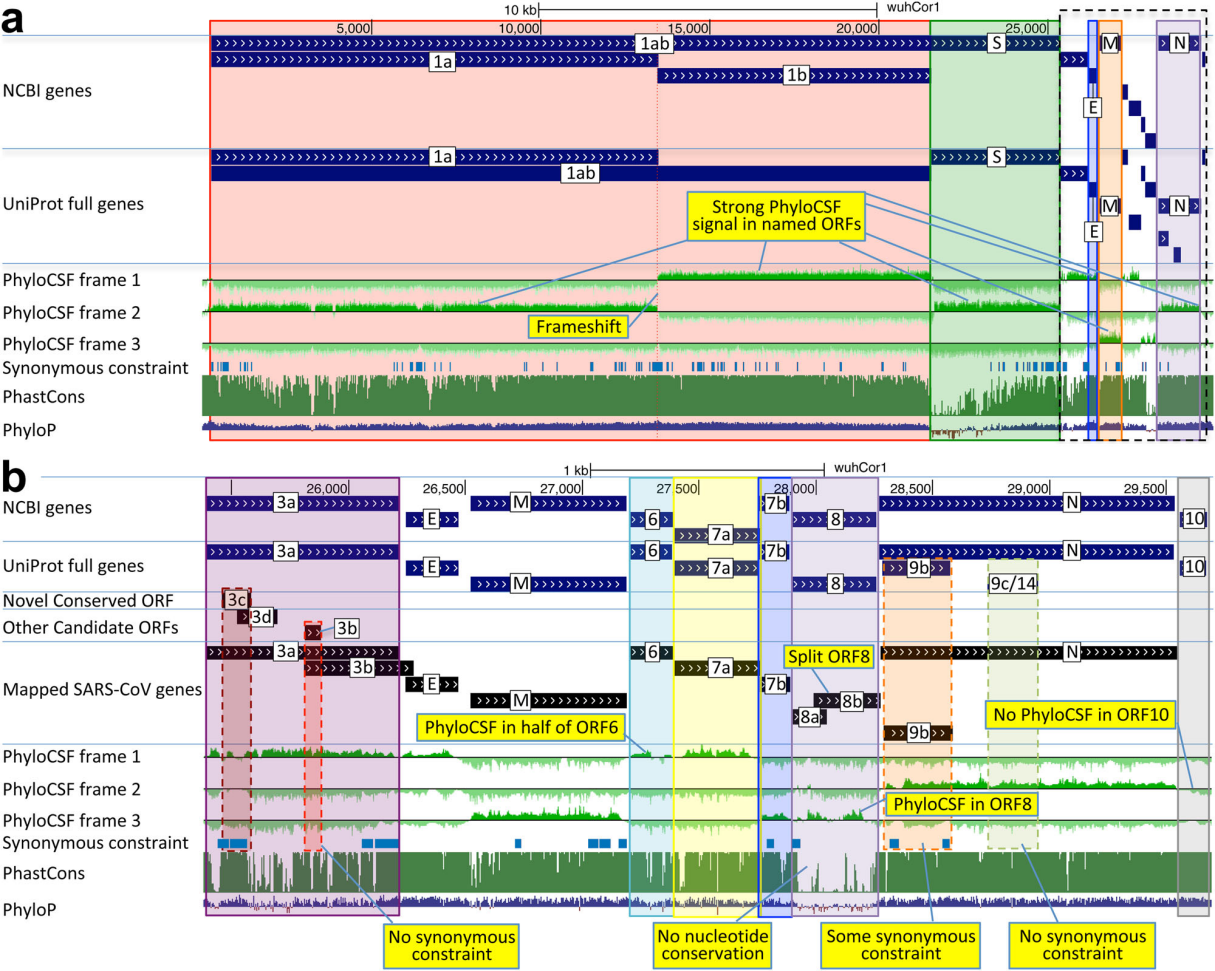
Genome-wide protein-coding signatures. SARS-CoV-2 NCBI/UniProt genes (blue), unannotated candidate genes and mapped SARS-CoV genes (black, panel **b** only), frame-specific protein-coding PhyloCSF scores (green), Synonymous Constraint Elements (SCEs) (blue), and phastCons/phyloP nucleotide-level constraint (green/blue/red) across genomic coordinates (x-axis) for entire genome (panel **a**) and final 4-kb subset (panel **b**, dashed black box): **a** strong protein-coding signal in correct frame for each named gene; conservation-signal frame-change at programmed frameshift site; strong protein-coding signal throughout S despite lack of nucleotide conservation in S1; **b** unambiguous and frame-specific protein-coding signal for ORFs 3a (despite only partial nucleotide conservation), 7a, 7b, and 8 (despite lack of nucleotide conservation); clear protein-coding signal in first half and last quarter of ORF6; no protein-coding signal for 10 (despite high nucleotide conservation); synonymous constraint (blue) in novel-ORF 3c and confirmed-ORF 9b; no synonymous constraint in rejected ORFs 9c, 3b, 3d.

SARS-CoV-2, the virus responsible for COVID-19^1^, is a member of the species *Severe acute respiratory syndrome-related coronavirus* in the family *Coronaviridae*, subfamily *Orthocoronavirinae*, genus *Betacoronavirus*, subgenus *Sarbecovirus*^2^. This species also includes SARS-CoV, the virus responsible for the 2003 SARS outbreak. The large 29,903-nucleotide positive-strand RNA genome of SARS-CoV-2 encodes ~30 known and candidate mature proteins (Figs. 1a, 2, and Supplementary Fig. 1). Despite SARS-CoV-2’s extreme medical importance, its gene content has not been fully resolved, with several open-reading frames (ORFs) whose function or even protein-coding status is unknown. Moreover, no systematic resource exists for interpreting the functional impact of SARS-CoV-2 mutations and prioritizing candidate drivers that may underlie phenotypic differences between strains.

SARS-CoV-2 includes the six ORFs that are common to all coronaviruses^3^. At the 5′ end are two large ORFs, ORF1a and ORF1b, covering more than two-thirds of the genome. Canonical translation of ORF1a yields polyprotein pp1a. Alternatively, a programmed −1 frameshift four codons before the end of ORF1a directs a proportion of ribosomes to continue translation in an alternate reading frame until the end of ORF1b, yielding polyprotein pp1ab^4^. The name ORF1ab is sometimes used to refer to the two ORFs combined via the frameshift. In most coronaviruses, polyproteins pp1a and pp1ab are proteolytically cleaved into 11 or 15 mature non-structural proteins (nsps), respectively, namely nsp1–11 for pp1a or nsp1–10 and nsp12–16 for pp1ab, though nsp1 is absent in genus *Gammacoronavirus*^3^. The 5′ ends of the genomic regions encoding nsp11 and nsp12 coincide, but the final four codons of nsp11 are translated in different reading frames, allowing translation of nsp12 to bypass the nsp11 stop codon and continue downstream. The functional domains of many of the nsps have been well characterized, including the 3C-like cysteine proteinase (3CL^pro^, nsp5), RNA-dependent RNA polymerase (RdRp, most of nsp12), nidovirus RdRp-associated nucleotidyltransferase (N terminal of nsp12), helicase (Hel, nsp13), and exonuclease (ExoN, nsp14)^5,6^. Other nsps are involved in host-cell suppression, immune suppression, and diverse viral functions (Supplementary Data 2)^3^. Nsps within ORF1a are largely responsible for control of genome expression and those within ORF1b for replication^7^.

The last third of the genome encodes four named proteins that are present in all coronaviruses, namely S (spike surface glycoprotein), composed of S1 (viral attachment to host-cell ACE2 receptor) and S2 (membrane fusion, viral entry), E (envelope protein), M (membrane glycoprotein), and N (nucleocapsid phosphoprotein, RNA genome packaging). Their host-cell translation requires subgenomic RNAs of varying lengths, such that each functional ORF is first (or early) on its own transcript^8^. These subgenomic RNAs result from synthesis of negative-sense intermediates by transcription starting from the 3′ end of the genomic RNA, extending to one of several internal transcription-regulatory sequences (TRS), and looping to a common 5′ leader sequence; the negative-sense intermediates are then used as templates for synthesis of positive-sense subgenomic RNAs^3,9^.

The last third of the genome also encodes several unnamed ORFs that are specific to the species *Severe acute respiratory syndrome-related coronavirus* or to the subgenus *Sarbecovirus*. These include five “accessory” ORFs previously identified in other viruses of the species, namely, from 5′ to 3′, ORFs 3a, 6, 7a, 7b, and 8 (split into ORF8a and ORF8b in some SARS-CoV isolates)^1,10,11^, and several others that are not universally annotated and are subject to disagreement on which encode functional proteins in SARS-CoV-2 (Supplementary Data 2). NCBI includes ORF10 in its reference annotations (NC_045512.2). UniProt also annotates ORFs 9b and 9c (which is also called 14), both overlapping N in an alternate frame. The paper introducing the SARS-CoV-2 genome also shows ORF3b (which overlaps ORF3a in SARS-CoV but is truncated in SARS-CoV-2, with several in-frame stop codons)^1^. Other publications^12–20^ include different subsets, use different names, or propose additional ORFs (including ORFs 3c, 3d, and 3d-2 overlapping ORF3a, and ORF2b overlapping S). NCBI annotates SARS-CoV (NC_004718.3) orthologs of ORFs 3a, 6, 7a, 7b, and 9b, but ORF8 is split into ORF8a and ORF8b, ORF3b is included, and neither ORF9c nor ORF10 are included. Here we use the homology-based ORF nomenclature^21^ discussed in Supplementary Note 1.

High-throughput experiments provide some evidence on SARS-CoV-2 gene content, though they sometimes disagree, cannot prove non-functionality of non-detected ORFs (as they only capture specific conditions), and cannot distinguish incidental transcriptional/translational events from selected function. Proteomics experiments identified peptides for ORFs 1ab, S, 3a, M, 6, 7a, 8, N, and 9b, but not E, 3b, 7b, 9c, or 10^22,23^. Direct-RNA sequencing found subgenomic RNAs for a different subset: S, 3a, E, M, 6, 7a, 7b, 8, and N, but limited or no support for 2b, 3c, 3d, 3b, 9b, 9c, and 10^23–26^, with 2b, 3c^16^, 7b^27^, and 9b^5^ possibly translated by leaky ribosomal scanning from S, 3a, 7a, and N subgenomic RNAs, respectively. Ribosome profiling predicted translation of 1ab, S, 3a, E, M, 6, 7a, 7b, 8, N, and 10, and eleven alternate-frame ORFs including 2b, 3c, 3d-2, and 9b, but not 3d, 3b, or 9c^20^.

In this work, we use comparative genomics of 44 *Sarbecovirus* strains to resolve the SARS-CoV-2 protein-coding gene set (Fig. 1), and to distinguish mutations more likely to have functional importance. We select 44 closely related and complete coronavirus genomes, generate whole-genome alignments, evaluate protein-coding and nucleotide-level constraint, and annotate synonymously constrained codons. We confirm that seven accessory ORFs encode conserved functional proteins, including novel alternate-frame ORF3c within ORF3a, and show that five candidates are not conserved and unlikely to encode functional proteins. We use protein-level and nucleotide-level inter-strain constraint to analyze 1875 mutations from 2544 pandemic isolates, show gene-level and codon-level agreement between within-strain and across-strain selective pressures, reveal recent adaptive acceleration for N and unexpected deceleration for S1 and nsp3, provide clues to the function of ORF8, and examine the evolutionary histories of spike-protein residues disrupted by mutations associated with increased transmission or immune evasion, and mutations co-inherited with them, to find clues about their biology. We also apply several measures of conservation to previously found RNA-modification sites and find no enrichment.

## Results

### What we mean by gene and ORF

In order to resolve the SARS-CoV-2 protein-coding gene set, we need to first clarify what we mean by ORF and protein-coding gene since the terms are used with slightly different meanings by different authors. Here, we use ORF to mean any contiguous stretch of codons beginning with a start codon, ending with a stop codon, and with no intermediate in-frame stop codons, though adjusting for the programmed frameshift in ORF1ab. We do not require an ORF to be translated or exceed any minimum length. It is standard in the bioinformatics community to define ORF in a way that does not require evidence of translation, though this definition might be less familiar in the virological community. We will only consider an ORF to be a “protein-coding gene” if it is translated into a *functional* protein, by which we mean a protein that contributes to viral transmission, replication, immune avoidance, or overall fitness. Translation is a necessary but not a sufficient condition for an ORF to be a protein-coding gene, since the act of translation can serve a function even if the peptide it produces is not functional, such as for regulatory uORFs^28^, and low levels of translation can result from random neutrally evolving sequence features without providing any fitness benefit to the virus. The requirement that the gene be functional at the protein level is common in eukaryotic gene annotation projects such as GENCODE. We recognize that this definition is a theoretical ideal, and that labeling an ORF as protein-coding or not must be considered tentative and subject to change as additional evidence accumulates. We note that a translated ORF can be important even if it is not a protein-coding gene if it encodes an antigen detectable by the immune system or a diagnostic test.

### Strain selection and alignment, constraint

**Fig. 3:**
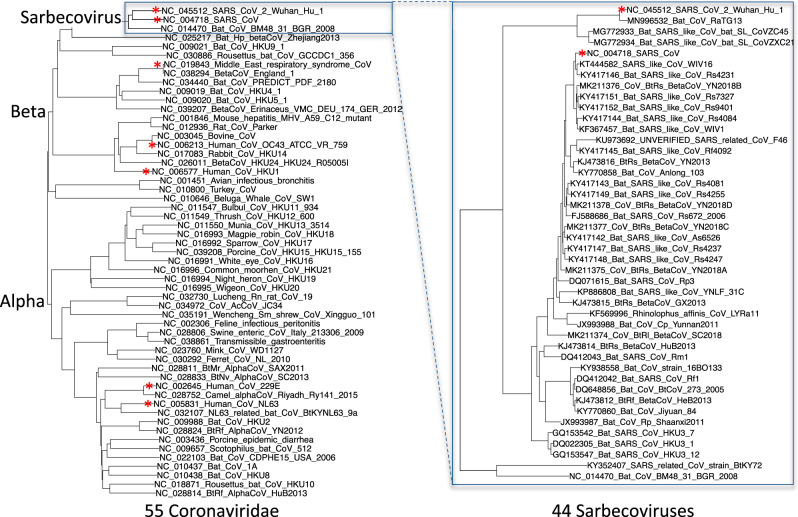
Phylogenetic tree of 44 *Sarbecovirus* genomes and larger phylogenetic context. Left: Phylogenetic tree of a selection of *Orthocoronavirinae* genomes, including the seven that infect humans (red asterisks). Right: Phylogenetic tree of the 44 *Sarbecovirus* genomes used in this study (all belong to the species *Severe acute respiratory syndrome-related coronavirus*). Trees are based on whole-genome alignments and might be different from the history at particular loci, due to recombination.

We selected and aligned 44 complete *Sarbecovirus* genomes (SARS-CoV-2, SARS-CoV, and 42 bat-infecting strains, Fig. 3, Supplementary Data 1) at evolutionary distances well-suited for identifying protein-coding genes and non-coding purifying selection, spanning ~3 substitutions per 4-fold degenerate site on average (comparable to 29-mammals/12-flies projects^29,30^), and ranging from 1.2 (E) to 4.8 (O-MT/nsp16) and higher (Supplementary Data 2). Betacoronaviruses outside *Sarbecovirus* (including MERS-CoV) are too distant (e.g. no detectable homology across ORFs 6-7a-7b-8), and SARS-CoV-2/SARS-CoV isolates are too proximal for reliable evolutionary signatures. Evolutionary distances between SARS-CoV-2 and other sarbecoviruses, as measured by nucleotide identity, vary substantially across the genome (Supplementary Fig. 2, Supplementary Data 9).

As of this writing, all known viruses in the subgenus *Sarbecovirus* belong to the species *Severe acute respiratory syndrome-related coronavirus*. Consequently, results reported here that are currently universal among known sarbecoviruses might or might not apply to other *Sarbecovirus* species discovered in the future. These taxonomic ranks were demarcated using *Coronaviridae*-wide criteria based on comparative sequence analysis using DEmARC software^31^. To put them in perspective with respect to clades that have been used previously for identifying protein-coding genes via evolutionary signatures, evolutionary distances measured by neutral substitutions per site within the species *Severe acute respiratory syndrome-related coronavirus* correspond roughly to those within the placental mammals infraclass and are somewhat less than those in genus *Drosophila*.

### Evolutionary signatures of protein-coding genes

To detect protein-coding evolutionary signatures and distinguish regions evolving under protein-coding constraint, we previously developed PhyloCSF^32^, which compares codon substitutions and frequencies in alignments of related genomes to coding and non-coding models of evolution trained on whole-genome data (Fig. 1b), and CodAlignView^33^, which facilitates visual examination of the corresponding alignment for substitutions, stop codons, insertions, and deletions indicative of protein-coding or non-coding status. These tools primarily exploit two main evolutionary signatures characteristic of protein-coding genes across evolutionary time: first, a preference for synonymous substitutions that preserve amino acid translation and conservative amino acid changes that preserve biophysical properties; second, avoidance of stop codons and insertions or deletions that are not multiples of three as they would disrupt the reading frame of translation. These tools are widely accepted standards for protein-coding gene annotation and for distinguishing protein-coding vs. non-coding genes in human and other species^29,30,32,34–36^, but have never before been applied to viruses.

We quantified protein-coding constraint by computing PhyloCSF scores for every three-nucleotide interval in all three reading frames of the SARS-CoV-2 genome, using our 44 *Sarbecovirus* whole genome alignments. We smoothed these scores using a hidden Markov model and created tracks for the UCSC Genome Browser^1,37,38^ (Fig. 2), as we previously did for the human and other genomes^34^. We also computed an overall PhyloCSF score for each known and candidate protein and mature product, and provide hyperlinks to visualize their alignments in CodAlignView for manual exploration in all reading frames (Supplementary Data 2, Fig. 1c).

We used FRESCo, a software tool we had previously developed and applied to diverse virus species^39^ and human^40^, to calculate the rate of synonymous substitutions in the alignment of each codon of the NCBI-annotated genes and to detect regions having significantly lower synonymous rate, indicating nucleotide-level constraint that goes beyond what is needed to preserve the amino acid sequence and is thus indicative of overlapping functional elements. Such elements can include: dual-coding regions when multiple proteins are encoded in different reading frames, RNA structures folding from stretches of complementary nucleotides and known to play important roles in subgenomic RNA generation and other coronavirus functions, and binding sites for RNA-binding proteins. FRESCo was used previously to find synonymous constraint elements (SCEs) in 30 species of viruses, including ones with double-stranded and single-stranded, plus and minus sense, segmented and unsegmented, DNA and RNA genomes, having plant, insect, and mammal hosts. It was validated using simulated data and by recovering known overlapping genes in a wide variety of viruses, and then predicted novel overlapping elements in other viruses, including putative RNA structural elements in foot-and-mouth disease virus, infectious bursal disease virus, potato virus Y, and turnip mosaic virus^39^.

We defined SCEs within each gene based on synonymous rates in 9-codon windows that are significantly decreased relative to the gene average^39,41^ resulting in 92 SCEs covering 1555 codons. We also annotated 1394 individual codons (14% of 9744) having substantially reduced synonymous rate (false discovery rate <0.125).

We also computed SCEs relative to the average synonymous rate within each nsp, since comparison to a local neighborhood is less likely to be biased by variations in mutation rate across the genome than comparison to the full gene (ORF1a or ORF1ab). The SCEs computed relative to each nsp are similar to those computed relative to the complete gene, and most differences are SCEs whose *p*-value is near our significance threshold. Nsp boundaries are not natural boundaries for SCE analysis because SCEs are RNA elements typically involved in regulating transcription, translation, and RNA processing, whereas nsps result from post-translational processing of the amino acid chain; in fact, an SCE that crosses the boundary between nsp4 and nsp5 is lost if these two are treated as separate genes. Consequently, we used the gene-wide SCEs for subsequent analyses but made both sets available in a track hub for the UCSC Genome Browser^42^.

**Fig. 4:**
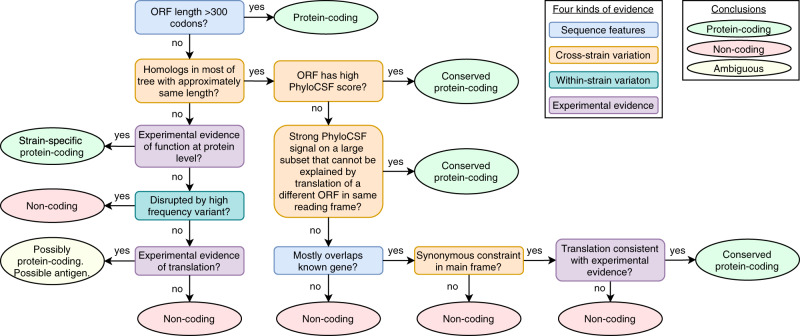
Protein-coding decision flow chart. Flow chart indicates main steps in determining if an ORF encodes a functional protein (light green ovals), is not protein-coding (red ovals), or is translated but with ambiguous protein-coding status (yellow oval), with cases for conserved non-overlapping, conserved overlapping, and non-conserved ORFs. Decisions are based on sequence features (blue rectangles), evolutionary signatures across *Sarbecovirus* (orange rectangles), within-strain variants (dark green rectangle), or experimental evidence (purple rectangles). Actual process considers additional details (Supplementary Note 2).

We use a multi-step decision process to distinguish functional protein-coding genes (Fig. 4, Supplementary Note 2). In brief, we use PhyloCSF to distinguish non-overlapping conserved ORFs, a combination of PhyloCSF and synonymous constraint to distinguish overlapping conserved ORFs, and rely primarily on experimental data to distinguish de novo ORFs in a lineage, though many other factors must be considered.

### Coding constraint on non-overlapping genes

As validation of our method, we see a clear PhyloCSF signal of protein-coding constraint extending the full length of each of the six coronavirus-wide ORFs (ORF1a, ORF1b, S, E, M, and N), including each of the nsps nsp1–10 and nsp12–16, with a change in constrained reading frame at the known programmed frameshift site (Supplementary Data 2, Fig. 2, Supplementary Fig. 1). Beyond its first 9 codons that match RdRp, the 13-codon nsp11 showed no nucleotide changes among our sarbecoviruses, but stop-codon gain/loss across beta coronaviruses suggests it is not separately functional (Supplementary Fig. 4). S1 shows extremely rapid nucleotide evolution (near-zero phyloP^43^ and phastCons^44^, Supplementary Data 2) but strong PhyloCSF scores, highlighting the power of PhyloCSF to recognize protein-coding evolutionary signatures despite rapid nucleotide evolution.

Among ORFs that have been previously described in some members of the species *Severe acute respiratory syndrome-related coronavirus*, ORFs 3a, 7a, 7b, and 8 show clear positive PhyloCSF scores, indicating that selection for protein-coding function has been present throughout all or most of the clade (Fig. 2b). The first half and last quarter of ORF6 show a strong PhyloCSF signal, indicating that it too encodes a conserved functional protein, despite a less-constrained intermediate portion, and an overall near-zero average score per codon (−0.3, Fig. 1c). ORF8 shows a strongly positive protein-coding PhyloCSF score (4.61/codon), and long stretches of strong protein-coding constraint, indicating unambiguous protein-coding function conserved through most of the clade, despite showing near-zero nucleotide-level conservation (phyloP/phastCons, Supplementary Data 2) and lacking well-established functions. Its high nucleotide-level rate is inflated by past recombination but remains high even using an ORF8-specific phylogeny (Supplementary Fig. 5).

**Fig. 5:**
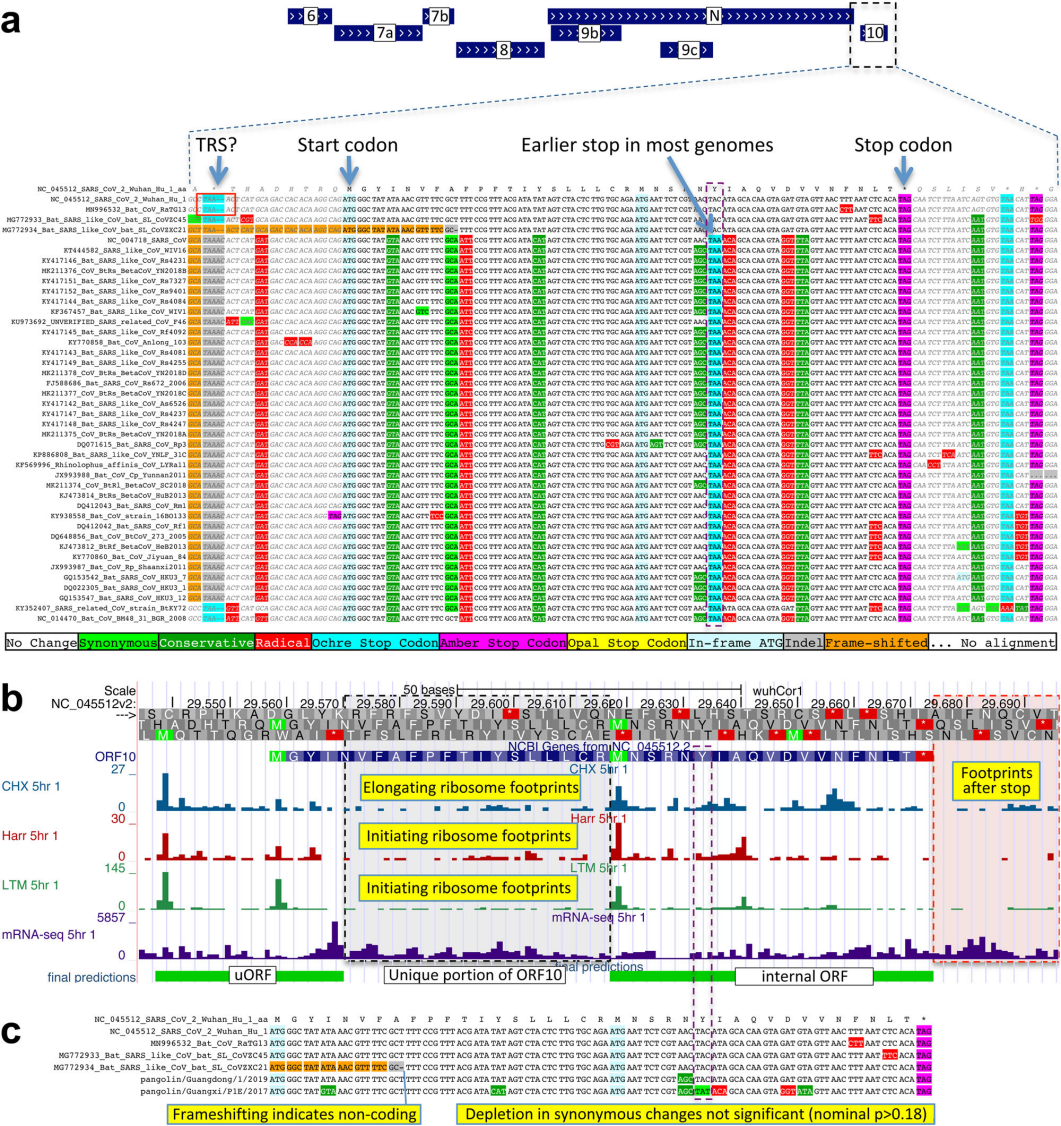
ORF10 is not protein-coding. **a** Alignment of *Sarbecovirus* genomes at ORF10, including 30nt on each side. Most substitutions are radical (red) or conservative (dark green) amino-acid-changing, with only two synonymously changing positions (light green), indicating this is not a conserved protein-coding ORF. Nearly all strains show an earlier stop codon (cyan), further reducing the length of this already-short ORF from 38 codons to 25, and another strain includes a frame-shifting deletion (orange). Putative partial transcription-regulatory sequence (TRS) present in SARS-CoV-2 and Bat CoV RaTG13 is not present in other strains. The surrounding region shows high nucleotide-level conservation, spanning ORF10 and extending beyond its boundaries in both directions, indicating this region is functionally important even though it does not encode protein (indeed, it is part of a known RNA structure). **b** Ribosome footprints previously used to suggest that ORF10 might be translated^20^ in fact localize either in an upstream ORF (uORF, green) or in an internal ORF (green, “final predictions” track^20^), but density in the unique portion of ORF10 (dashed black box) is no greater than after the stop codon (red box), indicating they are less likely to reflect the functional translation of ORF10, and more likely to represent incidental translation initiation events. The internal ORF is only 18 codons long in 4 strains, and 5 in the other strains, given the early stop codon (purple box), and unlikely to be functional. Footprint tracks show elongating ribosome footprints in cells treated with cycloheximide (blue, CHX), and footprints enriched for initiating ribosomes using harringtonine (Harr, red), and lactimidomycin (LTM, green). “mRNA-seq” track shows RNA-seq reads. **c** Alignment of six closely related strains (SARS-CoV-2, three bat viruses, two pangolin viruses) previously used to argue that high d*N*/d*S* ratio in ORF10 indicated positive selection for protein-coding-like rapid evolution^15^. A frameshifting deletion (orange/gray) in one bat virus militates against conserved protein-coding function. Even ignoring that strain, the evidence is not statistically significant: the alignment includes only 9 substitutions, including 1 synonymous. In a neutrally evolving region with 9 substitutions, we would expect 2–3 synonymous changes, and a depletion to only 1 is not statistically significant even without multiple-hypothesis correction (*P* > 0.18).

By contrast, ORF10 shows no protein-coding constraint anywhere along its length, contains in-frame stop codons in all but four sarbecoviruses truncating the last third of its already short length (38 codons), and includes a frame-shifting deletion in one of those four strains, indicating it is not protein-coding. Although it shows near-perfect nucleotide-level conservation (phyloP/phastCons), this extends beyond the ORF on both sides, indicating a non-coding function rather than protein translation (Figs. 2b and 5a). This region overlaps the 3′-UTR pseudoknot RNA structure^45^ involved in RNA synthesis, providing a likely explanation for its high nucleotide-level constraint. The alignment of ORF10 is strongly enriched for the combinatorial and spatial patterns characteristic of intergenic bases^46^, consistent with the non-coding status of ORF10. Moreover, ribosome footprints in the region occur in an overlapping upstream ORF or in a truncated ORF rather than uniquely in ORF10, consistent with incidental initiation events rather than functional translation (Fig. 5b), and previously used comparative evidence for protein-coding function ignored a frameshifting deletion and was insufficiently powered (Fig. 5c).

### N-overlapping ORF 9b is coding, 9c is not

Evolutionary evidence for overlapping ORFs is more difficult to resolve, as protein-coding signatures in the primary reading frame heavily influence scores in alternate frames. However, conservation of the alternate-frame amino acid sequence leads to a depletion of synonymous substitutions in the primary ORF localized over the overlapping segment, resulting in a strong signal of overlapping-constraint^39–41^ We next used this fact to investigate ORFs 9c and 9b overlapping N.

**Fig. 6:**
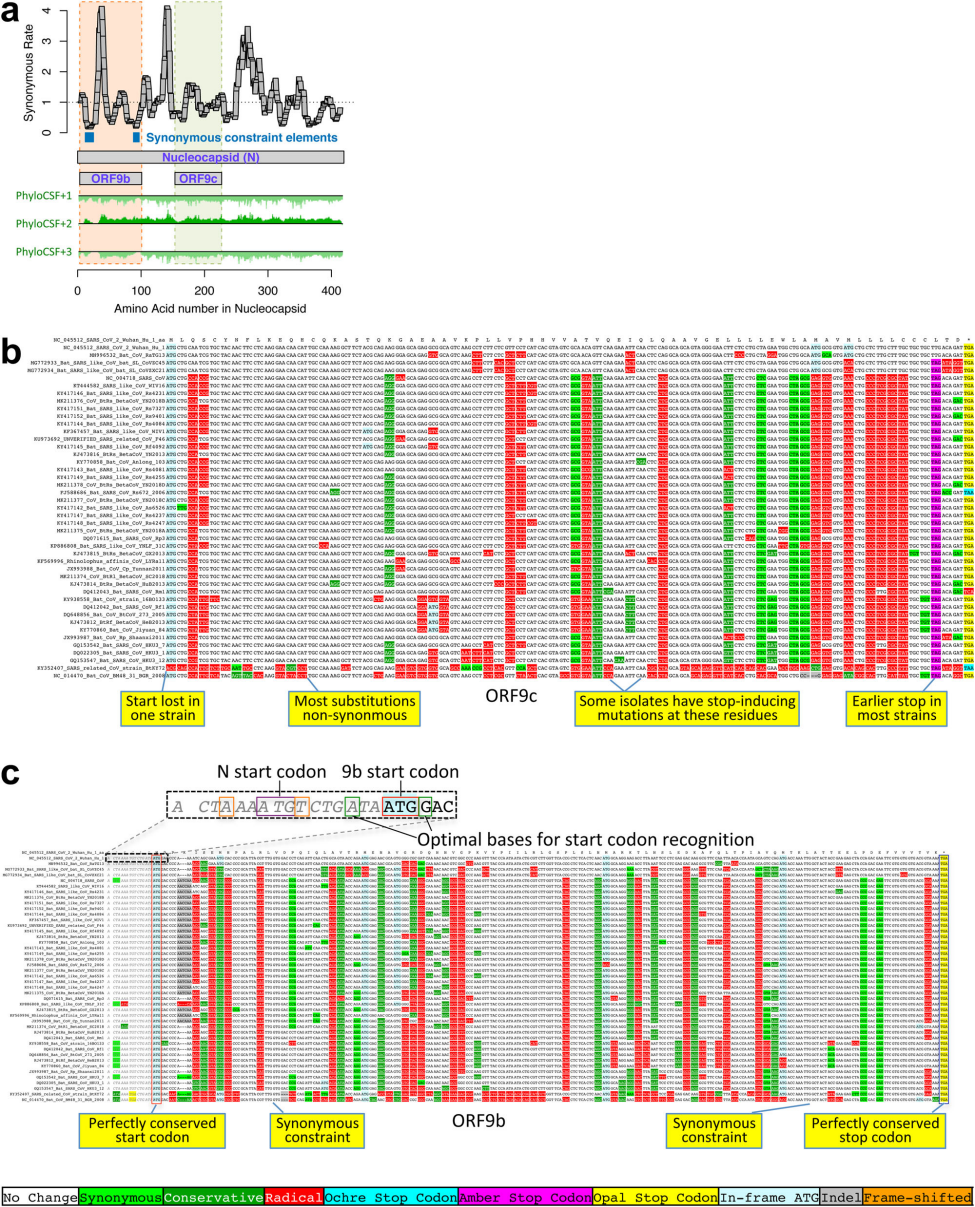
Nucleocapsid-overlapping ORF9b is protein-coding but not ORF9c. **a** Synonymous substitution rate in 9-codon windows (*y*-axis) across N (*x*-axis), normalized to gene-wide average (dotted black line). Two small synonymous constraint elements (SCEs, blue) expected for dual-coding regions localize near ends of overlapping 97-codon ORF9b (dashed orange rectangle), but the synonymous rate is high in the central portion. No SCEs localize to 73-codon ORF9c (dashed green rectangle). PhyloCSF protein-coding signal (green) in frame 3 (encoding ORF9b and ORF9c) remains strongly negative throughout ORF9c but rises to near-zero for two regions of ORF9b, while the N-encoding frame-2 signal remains consistently high throughout ORF9c. **b** *Sarbecovirus* alignment of ORF9c. Start codon is lost in one strain, and most have a UAG stop codon (magenta) 3 codons before the end. Nearly all substitutions are function-disrupting amino acid changes (red), and very few are synonymous (light green) or conservative (dark green), consistent with lack of PhyloCSF signal and synonymous constraint, indicating ORF9c does not play conserved protein-coding functions. Translation via leaky scanning is unlikely because ORF9c’s start is 460 nucleotides after N’s with 9 intervening AUGs (Supplementary Fig. 6), direct-RNA sequencing found no ORF9c-specific subgenomic RNAs^24–26^, and several SARS-CoV-2 isolates contain stop-introducing mutations^14^, indicating ORF9c is not a recently evolved strain-specific gene either. **c** *Sarbecovirus* alignment of ORF9b. Although ORF9b shows many function-disrupting substitutions, its start (red box) and stop codons (blue box) are perfectly conserved, with no intermediate stop codons in any strain. Its Kozak start-codon context (dashed black box) is optimal for ribosomal recognition (A/G in positions −3/+4, green boxes), while context of N is less optimal (A/T in positions −3/+4, orange boxes), with both contexts conserved across *Sarbecovirus* and no intervening AUGs, so ORF9b can be translated by leaky scanning from N’s subgenomic RNA. ORF9b has ribosome profiling^20^ and proteomics^22,23,49^ support in SARS-CoV-2, and experimental support in SARS-CoV^51–53^. Although high synonymous rate in N in central portion of ORF9b is unexpected for a dual coding region, synonymous constraint and near-zero PhyloCSF signal near its ends, and other evidence, suggest it is a conserved functional protein-coding gene, though one with high evolutionary rate in the central portion.

The 73-codon-long ORF9c (sometimes called ORF14 or ORF9b) shows no localized synonymous constraint in N (Fig. 6), calling its protein-coding status into question. A number of additional observations also suggest that ORF9c is not likely to be protein coding (Fig. 6, Supplementary Fig. 6): (1) its start codon is lost in one strain, (2) most strains have a three-codons-earlier stop, (3) its start codon is 460 nucleotides after N’s with 9 intervening AUG codons and thus unlikely to be translated via leaky ribosomal scanning, (4) direct-RNA sequencing found no ORF9c-specific subgenomic RNAs^24–26^ (and no TRS is appropriately positioned to create one), (5) neither ribosome footprint data^20^ nor proteomics evidence^22,23^ supports its translation, and (6) many SARS-CoV-2 isolates contain stop-introducing mutations in ORF9c^14^. Although two analyses found that ORF9c satisfied statistical tests for protein-coding regions based on single-genome data, neither compared to a null model to determine if the results were statistically significant^18,47^. ORF9c was found to suppress antiviral response in cells transfected with an expression ORF construct^48^, but there is no evidence that it is expressed from viral RNA during the course of infection. We conclude that ORF9c does not encode a functional protein.

The 97-codon-long ORF9b (sometimes called ORF9a) shows high synonymous substitution rate in N over much of the ORF but significant localized synonymous constraint in N for its start and end regions, even relative to the overall low synonymous rate of N (Fig. 6). This signal could arise from protein-coding constraint on parts of ORF9b if much of the protein were rapidly evolving, but could also result from SCEs unrelated to dual-frame coding if ORF9b were not protein-coding, so we looked to other evidence to resolve this ambiguity. The start and stop codons of ORF9b are perfectly conserved and its 97 codons are stop-free in all known sarbecoviruses. Its PhyloCSF score is negative, but this could be due to dual-coding signal biases. Its Kozak context is stronger than N’s and perfectly conserved and its start codon is only 10 nucleotides downstream of N’s, allowing it to be translated from N’s subgenomic RNA via leaky scanning (Fig. 6, Supplementary Fig. 7). ORF9b also has proteomics support^22,23,49^ (including evidence of viral-RNA binding^50^), and alternate-frame translation support by ribosome profiling^20^. In SARS-CoV, ORF9b protein (and antibodies to it) was detected in SARS patients^51,52^, localized in mitochondria, and interfered with host cell antiviral response when overexpressed^53^. On balance, this evidence suggests that ORF9b encodes a conserved functional protein, some portions of which are rapidly changing.

### ORF3c is a novel functional protein

We next searched for additional protein-coding genes by computing PhyloCSF scores for all 67 non-NCBI-annotated AUG-to-stop SARS-CoV-2 ORFs ≥25 codons long that are not contained in a longer same-frame ORF (locally maximal). None had positive PhyloCSF scores, but some may be coding as overlapping-ORF scores are reduced by alternate-frame protein-coding constraint, so we investigated near-zero top candidates for evidence of localized synonymous constraint, start/stop-codon conservation, and absence of in-frame stops or frameshifting indels.

**Fig. 7:**
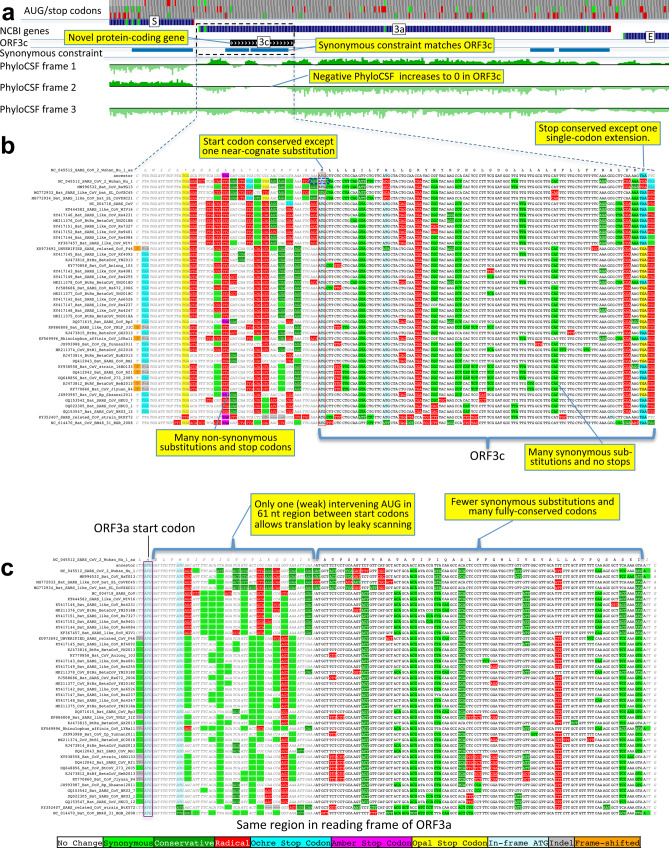
Novel gene 3c overlapping 3a is protein-coding. **a** Synonymous constraint elements (blue) match nearly perfectly 41-codon ORFc dual-coding region boundaries (black), and PhyloCSF protein-coding evolutionary signatures (green) switch between frame 1 and 2 (rows) in the dual-coding region, with frame-2 signal (negative flanking ORF3c) increasing to near-zero, and frame-1 signal (high flanking ORF3c) dropping to near-zero. **b**, **c** Codon-resolution evolutionary signatures (colors, CodAlignView^33^) annotating genomic alignment (letters) spanning ORF3a start and dual-coding region, in frame-1 (top) and frame-2 (bottom), highlighting (yellow boxes): (**b**, frame-2, ORF3c) radical codon substitutions (red) and stop codons (yellow, magenta, cyan) prior to ORF3c start; synonymous (light green) and conservative (dark green) substitutions in ORF3c; ORF3c’s start codon is conserved, except in one strain (row 4) with near-cognate GUG; ORF3c’s stop codon is conserved except for one-codon extension in two strains (rows 2–3); no intermediate stop codons in ORF3c; (**c**, frame-1, ORF3a) abundant synonymous and conservative substitutions in ORF3a prior to dual-coding region; increase in fully conserved codons (white) over dual-coding region indicating synonymous constraint. Short 61-nucleotide (nt) interval with only one weak-Kozak-context intervening start codon indicates ORF3c may be translated from ORF3a’s subgenomic RNA via leaky scanning.

The highest-scoring candidate, which we call ORF3c, overlaps ORF3a near its start (Fig. 7), with 38 of its 41 codons overlapping SCEs in ORF3a, localized nearly perfectly on the dual-coding region. Despite the score biases of dual-coding regions, ORF3c has a PhyloCSF score closer to non-overlapping protein-coding ORFs than to non-coding ORFs (Fig. 1c), indicating *Sarbecovirus* selection for protein-coding function. Strikingly, ORF3c also has many synonymous substitutions that are non-synonymous in ORF3a, indicating ORF3c may be an equally strong driver of constraint in the dual-coding region (both frames show similar scores in the dual-coding region). ORF3c also has conserved start and stop codons except for near-cognate GUG start in one strain and a one-codon extension in SARS-CoV-2 and RaTG13, with no in-frame stop codons or indels. We conclude ORF3c encodes a functional, conserved protein.

ORF3c was previously proposed (named ORF3h) using synonymous constraint across six closely related strains^15^ and a broader set of sarbecoviruses^16^, although on its own such evidence could also stem from other overlapping functional elements (and is abundant in SARS-CoV-2 even outside dual-coding regions), and using ribosome profiling (named 3a.iORF1)^20^, although such signal can also result from incidental, non-functional translation (and the other 8 such candidates lacked any conservation); it was predicted to contain a viroporin-like transmembrane domain^15^ and to be translated via leaky scanning^16^.

We examined all next-best-scoring candidates, and expanded the search to include shorter ORFs, near-cognate start codons, non-locally-maximal ORFs, and ORFs on the negative strand, but found no other convincing candidates (Supplementary Note 3, Supplementary Fig. 8, Supplementary Data 4), concluding that our catalog of conserved protein-coding genes is complete.

### ORFs 2b, 3d, 3d-2, and 3b are not conserved protein-coding ORFs

**Fig. 8:**
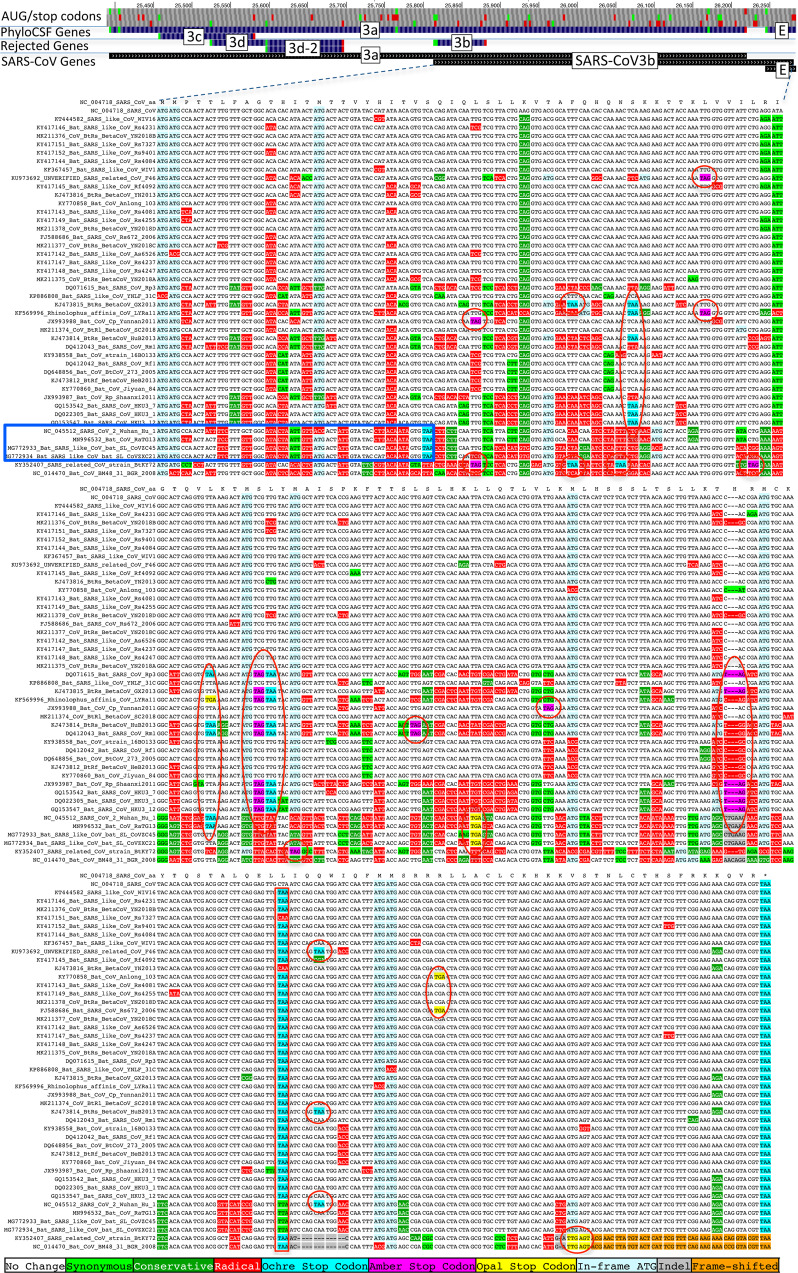
SARS-CoV-2 ORF3b is not protein-coding. *Sarbecovirus* alignment of SARS-CoV 154-codon ORF3b overlapping ORF3a (reordered with SARS-CoV and related strains on top). Although the start codon is conserved in all but one strain, ORF length is highly variable due to numerous in-frame stop codons (red ovals and red rectangle). The 22-codon ORF in SARS-CoV-2 has strongly negative PhyloCSF score, does not overlap any SCEs, and even among the four strains sharing its stop codon (blue rectangle) all six substitutions are radical amino acid changes, providing no evidence of amino-acid-level purifying selection. Ribosome profiling did not predict translation of ORF3b, transcription studies did not find substantial transcription of an ORF3b-specific subgenomic RNA, and translation by leaky scanning from the ORF3a subgenomic RNA would implausibly require ribosomal bypass of eight AUG codons (green rectangles, top panel), some with strong Kozak context. (Supplementary Fig. 9 has a comparison to the reading frame of ORF3a).

**Fig. 9:**
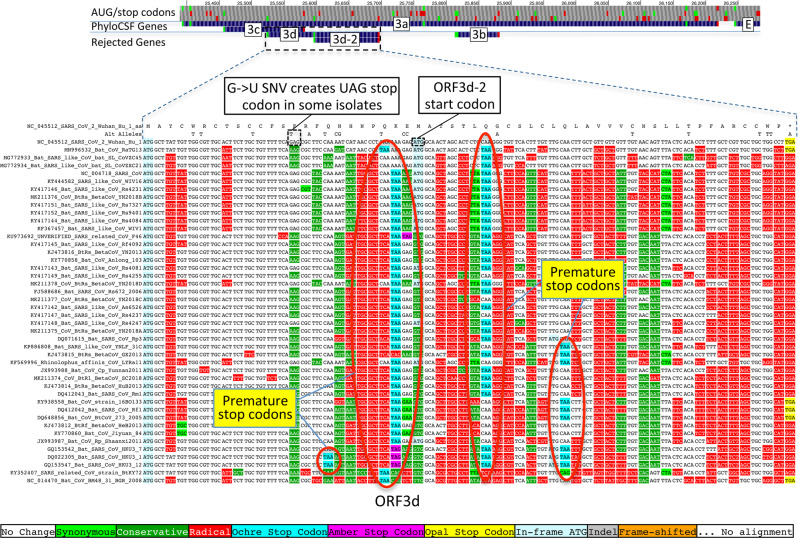
ORF3d is not protein-coding. *Sarbecovirus* alignment of 57-codon ORF3d (referred to by some authors as ORF3b) overlapping ORF3a shows mostly function-altering radical amino-acid substitutions (red columns), and repeated interruption by one or more premature stop codons in all other strains (red ovals), unambiguously indicating that ORF3d is not a conserved protein-coding gene. A substantial fraction of SARS-CoV-2 isolates have stop-introducing mutations, and ribosome profiling did not identify ORF3d as a translated ORF^20^, indicating that it is not a recently evolved strain-specific gene either. There is ribosome profiling and other evidence of translation of ORF3d-2, beginning at a downstream AUG and thus avoiding the stop-introducing mutations. However, ORF3d-2 is not conserved, is only 33 codons long, and lacks evidence that its translation product contributes to viral fitness.

Previous studies have proposed four other candidate protein-coding ORFs overlapping S and ORF3a, namely ORF2b (39 codons); ORF3d (57 codons); ORF3d-2 (33 codons), a subset of ORF3d starting at a downstream in-frame AUG; and ORF3b (22 codons), a truncated ortholog of SARS-CoV ORF3b^13,17–20^. (Note that these ORFs have been referred to by diverse names, with many papers referring to ORF3d as ORF3b^21^.) None of these are conserved in the species *Severe acute respiratory syndrome-related coronavirus*, showing non-conserved start codon, variable length, and premature stop codons. In fact, other than three closely related strains having same-length ORFs homologous to ORF3b (Fig. 8), none of these four ORFs have conserved homologs in any of the other 43 strains in our alignment (Fig. 9, Supplementary Fig. 10). Next, we consider whether these ORFs are newly protein-coding in SARS-CoV-2.

A ribosome profiling study predicted the translation of ORF2b^20^, and a proteomics experiment detected HLA-associated peptides from ORF2b^54^, providing evidence that ORF2b is translated but not that the resulting 39 amino-acid peptide is stable or functional.

The same ribosome profiling study also predicted translation of ORF3d-2 but not ORF3d^20^. Antibodies that react to a peptide translated from the ORF3d sequence were found in serum from former COVID-19 patients^55^, suggesting that ORF3d or its shorter isoform, ORF3d-2, is expressed at sufficient levels to generate an antibody response, but without distinguishing between the two or providing evidence that the protein contributes to viral fitness. ORF3d was found to have interferon antagonist properties when overexpressed from a plasmid^56^, but this is not evidence of translation from viral RNA during the course of infection. A comprehensive analysis reported several lines of evidence to suggest that ORF3d could encode a functional protein^17^, but each of them is ambiguous, not statistically significant, or cannot distinguish translation of ORF3d from translation of ORF3d-2 (Supplementary Note 4); in addition, a nonsense mutation, G25563U, that truncates ORF3d (but not ORF3d-2) has been found at substantial prevalence^14,17,56^, making it unlikely that translation of ORF3d contributes substantially to viral fitness.

ORF3b (22 codons) is orthologous to the 5′ end of SARS-CoV ORF3b, a 154 codon ORF whose various *Sarbecovirus* orthologs are truncated by numerous in-frame stop codons. Its start codon is conserved in all but one of our 44 *Sarbecovirus* strains, but its stop codon is only present in SARS-CoV-2 and its three closest relatives, and the ORF length is highly variable, so the SARS-CoV-2 form is not conserved (Fig. 8). The PhyloCSF score per codon of this truncated ORF is strongly negative (−18.0), it does not overlap any SCEs (Fig. 2b), and all six substitutions among the four closely related strains sharing this stop codon are radical amino acid changes, providing no evidence that this amino acid sequence has been under purifying selection. Overexpression in a human cell line of the SARS-CoV-2 ORF was found to have anti-IFN-I activity^19^, but this is not evidence of expression from viral RNA during the course of infection. SARS-CoV-2 ORF3b is extremely short; in fact, none of the 3054 viral proteins having protein-level evidence in the UniProtKB/Swiss-Prot database are as short as ORF3b. There is no TRS in the 5′ neighborhood of the ORF3b start codon, and in order for ORF3b to be translated by leaky scanning from the subgenomic RNA for ORF3a, the ribosome would have to bypass eight AUG codons, including several with moderate or strong Kozak context. It has been suggested that SARS-CoV ORF3b might be translated from an internal ribosomal entry site^5^, which is known to occur for some ORFs in certain other coronaviruses^57–60^, but to our knowledge no evidence of such a structure for ORF3b has been found. Finally, ribosome profiling and transcription studies did not find translation of ORF3b or substantial transcription of a subgenomic RNA from which it could be translated^20,23–25^.

We conclude that there is evidence that ORF2b and ORF3d-2 are translated, but no evidence that they encode functional proteins that contribute to viral fitness, and it is questionable whether ORF3d and ORF3b are translated at biologically meaningful levels.

### A new reference gene set for SARS-CoV-2

Altogether, our revised reference set of functional protein-coding genes consists of 1a, 1ab, S, 3a, 3c, E, M, 6, 7a, 7b, 8, N, and 9b, including novel ORF 3c and previously-ambiguous 9b, and excluding 3d, 3b, 9c, and 10. The genes in our reference set are unambiguously translated into conserved functional proteins across the species *Severe acute respiratory syndrome-related virus*, and our decisions are supported by a wealth of experimental evidence^20,22–26^, including subgenomic RNAs^23–26^ (or leaky scanning), ribosome profiling^20^, and proteomics experiments^22,23^ (Supplementary Note 5). Also excluded are 2b and 3d-2, which have evidence of translation but not of function. This high-confidence reference gene set can form the basis for understanding viral biology and the functional roles of pandemic mutations (Supplementary Note 6).

### *Sarbecovirus* conservation informs SARS-CoV-2 mutation impact

We next used the evolutionary history of each codon across sarbecoviruses to annotate 1875 single-nucleotide variants (SNVs) across 2544 SARS-CoV-2 isolates sequenced during the current COVID-19 pandemic, including 1142 amino-acid-changing (missense), 628 amino-acid-preserving (synonymous), and 104 non-coding mutations (Supplementary Data 3).

We classified all amino acid positions in each of the mature proteins and known or candidate protein-coding ORFs as “conserved” (no change in any of the 44 *Sarbecovirus* genomes) or “non-conserved/changed” (at least one change)(Supplementary Data 2), a definition independent of the phylogenetic tree, and thus resilient to recombination events common in coronavirus phylogenies^61^.

### Within-strain vs. cross-strains evolution

**Fig. 10:**
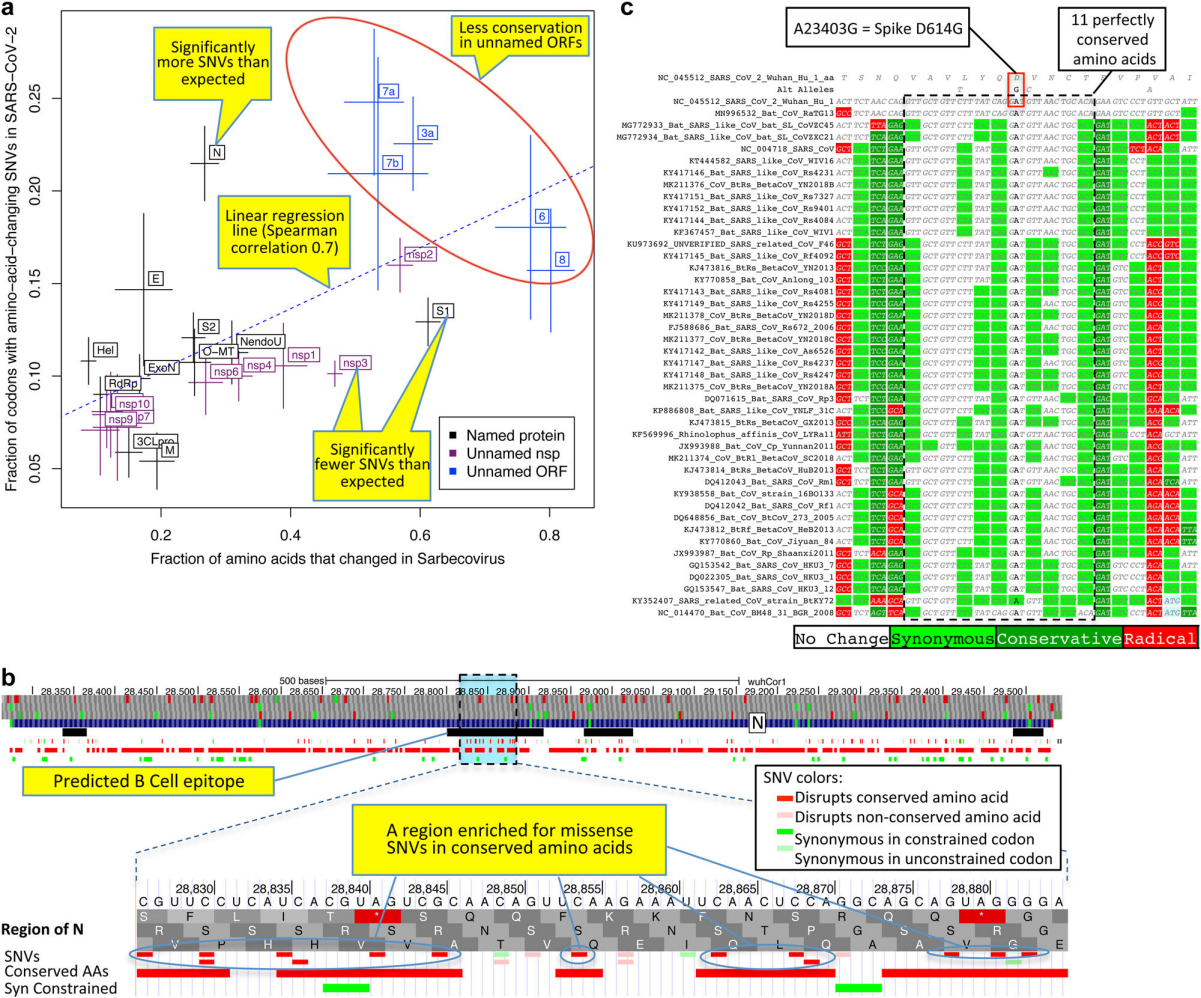
Within-strain variation vs. inter-strain divergence. **a** Gene-level comparison. Long-term inter-strain evolutionary divergence (*x*-axis) and short-term within-strain variation (*y*-axis) show strong agreement (linear regression dotted line, Spearman-correlation = 0.70) across mature proteins (crosses, denoting standard error of mean on each axis), indicating that *Sarbecovirus*-clade selective pressures persist in the current pandemic. Well-characterized coronavirus-wide genes (black) show fewer changes in both timescales (bottom left) and less-well-characterized ORFs (blue) show more in both (top right). Significantly deviating exceptions are: nsp3 and S1 (bottom right) showing significantly-fewer amino-acid-changing SNVs than expected from their cross-*Sarbecovirus* rapid evolution, and N (top left), showing significantly-more, possibly due to accelerated evolution in the current pandemic. **b** Rapidly evolving nucleocapsid region. Top: nucleocapsid-gene context showing B-cell epitope predictions (black, “IEDB Predictions” track), and our annotation track-hub showing: conserved amino acids (red blocks), synonymously constrained codons (green blocks), and SNV classification (colored tick-marks) as conserved/non-conserved (dark/light) and missense/synonymous (red/green); top 3 tracks show AUG codons (green) and stop codons (red) in three frames. Bottom: Focus on 20-amino-acid region R185-G204 (dotted box) in predicted B-cell epitope (black) significantly enriched for amino-acid-changing mutations (red) disrupting perfectly conserved residues, indicative of positive selection in SARS-CoV-2 for immune system avoidance. **c** Spike D614G evolutionary context. *Sarbecovirus* alignment (text) surrounding spike-protein D614G amino-acid-changing SNV, which rose in frequency in multiple geographic locations suggesting increased transmissibility. This A-to-G SNV disrupts a perfectly conserved nucleotide (bold font, A-to-G), which disrupts a perfectly conserved amino-acid (red box, D-to-G), in a perfectly conserved 11-amino-acid region (dotted black box, light-green = synonymous-substitutions) across bat-host sarbecoviruses, suggesting D614G might represent a human-host-adaptive mutation.

The fraction of changed amino acids varied greatly across ORFs (17–80%, Fig. 10a, *x*-axis), indicating dramatically different evolutionary pressures. Unnamed accessory ORFs had more changed amino acids (average 57%) than named and well-characterized ORFs (average 28%). ORF1ab mature proteins varied from 57% changed (nsp2) to <17% (3CL^pro^, RdRp, Hel, ExoN, nsp7–10) and spike-protein subunits from 61% changed (S1) to 25% (S2).

Faster-evolving proteins across sarbecoviruses showed more amino-acid-changing mutations within SARS-CoV-2 (Spearman correlation 0.70), indicating *Sarbecovirus* evolutionary pressures still apply during the current pandemic (Fig. 10a). This inter-vs.-within-strain agreement also held at codon resolution, with amino-acid-changing mutations preferentially disrupting non-conserved residues (535 mutations in 3264 positions, 16.4%) vs. conserved residues (607 in 6480, 9.4%, *P* < 10^−10^) (Supplementary Fig. 12a).

### Accelerated and decelerated evolution

Notable deviations from this general agreement may reflect recent accelerated/decelerated evolution. S1 showed significantly fewer mutations than expected from its extremely high inter-strain rate (13% amino-acid-changing mutations observed vs. 17% expected, nominal *P* = 0.0017, depletion: 28); additional SNVs (*n* = 2696, May 9, 2020) further strengthened the statistical significance of this result (*P* = 0.00033). Nsp3 also showed significantly fewer mutations than expected (10% vs. 15%, nominal *P* < 10^−9^, depletion: 90) and N significantly more (21% vs. 11%, nominal *P* < 10^−8^, excess: 42).

The lower-than-expected number of mutations in S1/nsp3 might indicate recent mutation-rate or selective-pressure changes, possibly stemming from different phases of host-adaptive evolution, with pre-pandemic earlier-adapting S1/nsp3 (eg. via non-human-host transmission or undetected human transmission) requiring fewer pandemic-phase human-adaptive mutations than other later-adapting genes (noting that only a subset of mutations are adaptive). Alternatively, S1/nsp3 may have more positions in which deleterious mutations would be strongly-deleterious (purified-out even in shorter timescales) vs. mildly-deleterious (purified-out only over larger timescales). Lastly, frequent S1 recombination could inflate inter-strain rate estimates, but probably insufficiently to account for the observed discrepancies. (Supplementary Note 7).

The higher-than-expected number of mutations in N might be explained by positive selection for host adaptation. We investigated whether such positively-selected variation might be clustered in specific segments, and searched the entire genome for clusters of mutations disrupting conserved amino acid residues. We found no significantly-depleted regions (Supplementary Note 8, Supplementary Fig. 11) and only one region significantly-enriched relative to gene-specific mutation density (*P* < 0.012 after conservative genome-wide multiple-hypothesis correction), which was indeed localized in N, and contained 14 mutations disrupting conserved residues (out of the observed excess of 29 such mutations in N) concentrated in 20-amino-acid region R185-G204 (noting this enrichment is relative to the already-high enrichment of such mutations in N). This region overlaps a predicted B-Cell epitope^62^, suggesting positive selection for immune system avoidance (Fig. 10b, Supplementary Fig. 12c).

### Spike SNV prioritization

We next used the evolutionary history of each amino acid across our 44 sarbecoviruses to provide position-specific estimates of evolutionary constraint for the SNVs defining SARS-CoV-2 lineages associated with phenotypic differences in order to determine which are most likely to be biologically relevant, thus taking into account the biological context and precise functions that each amino acid plays in coronavirus biology (beyond position-independent general estimates from general amino acid properties).

We first investigated 16 amino-acid-changing mutations in the spike gene that achieved high frequency during the spring of 2020 and/or had epitope proximity^63,64^ (Supplementary Data 3). Among them, radical-amino-acid-change D614G, which rose in frequency across multiple cities and increases infectivity in vitro^64–68^, disrupts a residue that is perfectly conserved among our 44 sarbecoviruses, and lies in a stretch of 11 otherwise perfectly conserved amino acids (Fig. 10c), indicating that its disruption is likely to be deleterious in bat-hosted viruses. We considered three possible explanations for the opposing fitness effects of this mutation in the SARS-CoV-2 pandemic versus in bat-hosted viruses. First, it could represent a novel human-host adaptation. However, the D614G substitution also increases SARS-CoV-2 infectivity in Chinese rufous horseshoe bats and Malayan pangolin cells^65^, suggesting that host differences alone are not sufficient to explain the discrepancy. Second, it could be due to a difference between SARS-CoV-2 and bat-hosted viruses, such as the furin cleavage site at the S1–S2 junction, which is unique to SARS-CoV-2^69^. A third possible explanation is that the D614G mutation biases the spike protein towards its ACE2-binding-competent state, making cell fusion more likely but possibly making the protein more susceptible to antibodies; in that case, the mutation might offer a fitness advantage through increased transmission in an immunologically naive host population, but then shift to a disadvantage once most potential hosts have been previously exposed^65^. Of the other 15 spike-gene mutations, two are in perfectly conserved residues (V615I/F, P1263L) and two in mostly conserved residues in highly conserved regions (A831V, A829T/S), indicating likely functional changes. Another three are in moderately conserved contexts (V367F, D839Y/N/E, D936Y/H) less likely to be functional, and eight lie in repeatedly-altered amino acids in poorly conserved regions and are more likely to be neutral.

We next investigated three amino acid substitutions in the receptor binding domain of the spike protein that have arisen repeatedly and are thought to increase infectivity or contribute to immune system avoidance. The B.1.1.7 lineage, which rapidly rose in frequency in the United Kingdom^70–72^, includes spike-protein substitution N501Y, which was found to increase ACE2-binding affinity^73,74^ and is thought to be responsible for the increased infectivity. N501Y arose, apparently independently, in the B.1.153 lineage, which rapidly rose in frequency in South Africa and also includes spike-protein substitutions E484K and K417N^75^, which are thought to decrease binding of antibodies from monoclonal antibody cocktails or from immune response to vaccines or previous infection with the wild-type virus^76,77^. Substitutions in these same three residues (but with K417 changing to T rather than N) arose independently in the P.1 lineage, which rapidly rose in frequency in some regions of Brazil^78,79^. E484K arose independently in another lineage, P.2, also found in Brazil^78^. Two of these three substitutions, E484K and N501Y, affect contact residues within the receptor-binding motif, the main functional motif that forms the interface with the human ACE2 receptor, and show evidence of positive selection in the SARS-CoV-2 population based on excess of non-synonymous substitutions and increasing frequency^75^. None of these three substitutions affect conserved residues (Supplementary Fig. 14a–c), showing that although *Sarbecovirus* conservation implies function, there are special cases for which the converse is not true. In particular, functional residues will not necessarily be conserved if they have been under positive selection through much of the *Sarbecovirus* tree, or if they are functional in SARS-CoV-2 but evolved neutrally in other strains. The regions around E484K and N501Y are highly variable among sarbecoviruses, containing many non-synonymous amino acid substitutions and, in the case of E484K, indels, consistent with the observed positive selection in SARS-CoV-2. In contrast, K417 is perfectly conserved among sarbecoviruses, except in the clade containing SARS-CoV-2 and closely related bat virus RaTG13 where the ancestral valine codon changed to lysine, in a string of 9 amino acids that are otherwise perfectly conserved. The high conservation of this region in the other 42 strains suggests this residue is functional, but might have changed to a non-optimal amino acid in the ancestor of the SARS-CoV-2/RaTG13 clade, perhaps due to drift, in which case this residue could be less constrained in SARS-CoV-2 and more likely to vary as a means to escape antibodies generated against the wild type virus.

Although these particular spike-protein substitutions are thought to be the main drivers of the distinguishing phenotypes of these lineages, they are co-inherited with additional mutations in each lineage. The B.1.1.7, B.1.153, and P.1 lineages have an unusually large number of co-inherited mutations, particularly amino-acid-changing spike-gene mutations^70,75,78^, possibly due to within-host evolution in an immunocompromised individual, which can accelerate the accumulation of mutations^80–82^. The B.1.1.7 lineage includes a 2-amino acid deletion S:del69-70 that causes S gene target failure in some PCR assays^83^, making the variant easier to detect, and that arose independently in an immune-suppressed individual treated with convalescent plasma^82^. We next examined the *Sarbecovirus* evolutionary context for each of the mutations co-inherited with any of D614G, N501Y, E484K, and K417N/T to determine those most likely to have some functional effect.

Spike-protein D614G was nearly always co-inherited with RdRp P4715L (also radical and altering a perfectly conserved residue in a highly conserved context, but potentially deleterious given RdRp’s slow evolution and less-likely-to-be-adaptive function), nsp3 nucleotide change C3037T (repeatedly observed synonymous change, outside synonymously constrained elements, likely neutral), and nucleotide change C241T (perfectly conserved, non-coding, in a loop of six unpaired bases in the conserved 5′-UTR SL5B secondary structure^45^ 25 nucleotides upstream of ORF1a).

We classified the 75 mutations that distinguish the B.1.1.7, B.1.153, P.1, and P.2 lineages relative to their respective parent lineages, including 69 SNVs, four deletions, and two multi-nucleotide substitutions, of which 17 disrupt conserved amino acids and one is a synonymous mutation in a synonymously constrained codon (Supplementary Data 15). Many of these conserved residues are in highly conserved regions of the protein, indicating that these mutations are very likely to have a functional impact. For example, the B.1.1.7 lineage includes mutations C5388A (orf1ab:A1708D) in a string of 7 perfectly conserved amino acids in a well-conserved region of nsp3, C14676T, a synonymous change in a large SCE in RdRp (situated between two conserved structures predicted by RNAz^45^ so possibly part of a containing structure too large for the prediction algorithm), T24506G (spike:S982A) in an extremely well-conserved region of S2, a three-nucleotide mutation at position 28280 (nucleocapsid:D3L) which weakens the initiation context of ORF9b, and C27972T (ORF8:Q27*) which truncates and presumably inactivates ORF8, which we discuss in more detail below; B.1.351 includes A10323G (orf1ab:K3353R) in a moderately conserved region of 3CL^pro^, G25563T (ORF3a:Q57H) which introduces radical amino changes in both ORF3a and ORF3c, and G13843T (orf1ab:D4527Y, present in about half of B.1.351 isolates) in a string of 33 perfectly conserved amino acids in RdRp; and finally, P1 includes G17259T (orf1ab:E5665D) in an extremely well-conserved region of Hel and C24642T (spike:T1027I) in string of 13 perfectly conserved amino acids in S2 (Supplementary Fig. 14d).

We conclude that many of the mutations co-inherited in these lineages are likely to have biologically meaningful effects, and may be contributing to the observed phenotypic consequences.

### Synonymous and non-coding mutations

Even for synonymous SNVs we found agreement between cross-strain and within-strain constraint, with synonymously constrained codons showing fewer synonymous mutations (73 of 1394, 5.2%) than non-synonymously constrained codons (555 of 8350 positions, 6.6%, binomial *P* = 0.029, Supplementary Fig. 12b).

We also classified 643 intergenic and 5′/3′-UTR positions as “conserved” (*n* = 432, 67%) or “non-conserved” (Supplementary Data 3), and found a surprising (but non-significant) SNV excess in conserved positions (17.4% vs. 13.7%, *P* = 0.17).

### RNA modification sites are not conserved

We next investigated the conservation of 83 RNA modification sites previously reported in two studies, 42 having 5-methylcytosine (m^5^C) modifications^25^ and 41 having RNA modifications of unidentified type^24^. RNA modifications such as m^5^C and N6-methyladenosine (m^6^A) can be detected using direct RNA sequencing^84^, and are known to play a role in regulation of replication and packaging of RNA viruses, as well as host response^85,86^.

If a specific RNA modification site serves a conserved function in *Sarbecovirus*, we would expect to observe excess constraint on both the site and its immediate context, which encodes motifs and RNA structures involved in its recognition by RNA modification enzymes^87^. We classified each RNA modification site according to whether it lies in a conserved nucleotide, an SCE, a conserved amino acid, or a synonymously constrained codon (Supplementary Data 5). We found no significant enrichment for conservation of the sites in either of these studies or in the combination of the two studies by any of these measures of conservation, even without multiple hypothesis correction (Supplementary Data 6). We also did not find these sites to be significantly depleted for SNVs relative to other sites that have matching base composition. The modified sites were strongly biased towards the 3′ end of the genome, with 92% being 3′ of the end of the S gene, so we repeated our calculations for the subset of the genome 3′ of S and again did not find significant enrichment for conservation according to any of our measures after multiple hypothesis correction, or significant depletion of SNVs.

Our enrichment analysis suggests that most RNA modification sites in SARS-CoV-2 do not serve a conserved function in *Sarbecovirus*, consistent with a previous study that found that most m^6^A modifications in mammals and yeast are non-functional and not conserved, probably resulting from off-target activities of m^6^A methyltransferases^88^. Our classification can help identify the subset of RNA modifications that are functional, since they are more likely to be conserved.

### ORF8 likely contributes to within-individual fitness but not transmission

We next investigated the truncation of ORF8 by the mutation ORF8:Q27* (C27972T), which changes a CAA sense codon to a UAA stop codon in the rapidly spreading B.1.1.7 SARS-CoV-2 lineage. The truncated ORF is only 27 codons long and presumably non-functional, indicating that ORF8 is not essential for SARS-CoV-2. However, as noted above, ORF8 shows strong evolutionary evidence of protein-coding function across this coronavirus species and experimental evidence of expression in SARS-CoV-2, together indicating that ORF8 loss would be expected to have a fitness cost, which is only tolerated due to hitchhiking with the highly advantageous N501Y spike protein substitution and possibly additional selected variants in the haplotype. We reasoned that nonsense-to-sense reversion of ORF8:Q27* may provide a further fitness benefit for B.1.1.7, and searched for isolates containing such events. Indeed, among the 49,675 non-degenerate B.1.1.7 genomes in GISAID (2021-Feb-05), 14 show UAA-to-CAA stop codon reversion, in at least seven independent reversion events (9 of the 14 result in full-length restoration to 121 or 126 codons, and 5 in partial restoration to 67 or 87 codons). These likely represent positive selection, as only 6 other isolates show mutations affecting the 15-base neighborhood surrounding Q27* (2 deletions and 4 SNVs of 3 distinct nucleotides), indicating that 7 distinct *27Q reversions are unlikely by chance. Surprisingly however, despite having at least 14 different opportunities to spread, in 10 different countries, these reversion events have not become a substantial fraction of all B.1.1.7 samples (representing only 0.03% of the 49,675 sequenced isolates).

To reconcile these seemingly conflicting observations of positive selection in 14 examples, but no increased transmission, we postulate that ORF8 may be advantageous for functions within an individual (e.g. viral replication and immune evasion), but neutral or even disadvantageous for transmission. This positive selection for an intact ORF8 within an infected individual would explain the frequent nonsense-to-sense reversions observed, and the non-advantage (or even disadvantage) of an intact ORF8 for transmission would explain the lack of substantial expansion of the reverted variants in each population where they occurred. Our hypothesis is consistent with the observation that ORF8 loss, which was observed in the SARS 2003 pandemic^89^, significantly decreases the rate of SARS-CoV viral replication in primate, bat, and human cell cultures^90^. Indeed, higher viral replication would provide an advantage for viral variants competing with other variants in the same infected individual, but its spread in the population would depend on how this increased replication rate affects host behavior and the period of contagion, and this effect may be neutral, or even detrimental. For example, increased replication may incapacitate the host more rapidly, may make an infection more easily detectable by the carrier and by others in the community, may shorten the asymptomatic period, may decrease the period of contagion, possibly by increasing immunogenicity or the speed of immune response. Differences in host behavior between humans and bats (e.g. wearing masks, avoiding crowds, staying home from work) could reconcile the apparent lack of overall fitness contribution of ORF8 in humans with the strong evolutionary evidence of selection for protein-coding function among the bat viruses in the rest of the *Sarbecovirus* clade. SARS-CoV-2 ORF8 might also contribute to immune avoidance by interfering with host MHC-1 molecules^91^, which might have weaker effect early in a pandemic while the host population is immunologically naive, so it is possible that ORF8 will make a larger contribution to overall viral fitness after most humans have been exposed to SARS-CoV-2.

## Discussion

We used comparative genomics to determine the conserved functional protein-coding genes of SARS-CoV-2, resulting in a new high-confidence evolutionarily and experimentally supported reference gene set, including ORFs 1a, 1ab, S, 3a, 3c, E, M, 6, 7a, 7b, 8, N, and 9b, but excluding 3d, 3b, 9c, and 10, which lack evidence of translation, and 2b and 3d-2, which lack evidence of function. We showed that novel ORF 3c is functional and conserved, and that no other conserved genes remain to be discovered.

Our comparative genomics evidence complements experimental approaches by providing a comprehensive function-centric view of protein constraint, summed over all environmental conditions and hosts spanned by the strains compared here, while experimental methods only profile a single environmental and host condition in each experiment. Moreover, while experimental methods can suffer from incidental transcriptional or translational events, evolutionary signatures specifically measure functional constraint for a given function. While in principle our methods may miss recently evolved genes that only function in a subset of strains, the lack of experimental evidence for ORFs other than those considered here suggests it is unlikely that we have missed any newly-evolved genes.

It is important to note that comparative genomics methods that focus on nucleotide-level constraint such as phyloP and phastCons, as valuable as they are, would have mistakenly rejected S1 and ORF8 as seemingly non-conserved (given their extremely-rapid evolutionary rate and recombination history), and conversely included ORF10 as seemingly conserved (given high nucleotide-level conservation in the overlapping RNA structure). Instead, our methods were able to correctly distinguish the protein-coding status of these genes because they use protein-coding evolutionary signatures that: (a) focus on the patterns of change characteristic of protein-coding constraint (specific codon substitution frequencies and reading frame conservation) rather than the overall number of substitutions; and (b) are less sensitive to the specific phylogenetic tree relating the genomes compared, and thus resilient to the recombination events that characterize coronavirus genomes.

We found that both protein-coding and non-coding constraint agree between cross-strain *Sarbecovirus* substitutions and within-strain SARS-CoV-2 mutations, enabling us to classify SARS-CoV-2 mutations into likely-functional vs. likely-neutral according to their evolutionary constraint. *Sarbecovirus* evolutionary histories provided clues to the biology of spike-gene mutations D614G, N501Y, E484K, and K417N/T and allowed us to catalog co-inherited mutations likely to have functional consequences. Beyond the specific examples cited here, our annotations are broadly useful for interpreting SARS-CoV-2 mutations and inferring causal relationships between viral mutations and disease phenotype. For interpreting future mutations, we also created a genome browser track hub to facilitate SARS-CoV-2 mutation interpretation based on their evolutionary context and based on our revised gene annotations.

We found three notable exceptions to the otherwise-strong agreement between inter-strain and within-strain variation: N showed significantly more amino-acid-changing mutations than expected, and nsp3 and S1 showed significantly fewer. For N, the acceleration is consistent with positive selection for human-host adaptation across many mutations, including a 20-amino-acid region enriched for conserved-residue-disrupting mutations in a predicted B-cell epitope. For nsp3 and S1, the deviation raises the possibility they may represent pioneer proteins that adapt to new-host transmission prior to its pandemic phase, then require fewer mutations while other proteins ‘catch up’, an observation that may be more generally true across different proteins showing acceleration/deceleration in different phases of host adaptation and pandemic spread. Another possibility is that the space of deleteriousness across all possible mutations is differently distributed for nsp3 and S1 compared to other proteins, with more deleterious mutations in the strongly deleterious end of the distribution, thus explaining the discrepancy in the number of observed amino acid substitutions between the short timescales captured in the recent pandemic SNVs vs. the longer timescales captured in cross-*Sarbecovirus* comparative genomics. We discuss these and other possibilities in Supplementary Note 7.

Although PhyloCSF and CodAlignView have been widely used for gene annotation and for the discovery of novel and unusual protein-coding regions in eukaryotic genomes, this is the first time these tools have been applied to a viral genome. Similarly, this is the first time FRESCo has been applied to help classify mutations within a viral strain. Our tools and workflow should prove useful for similar analyses in diverse species from across viral realms as more strains and isolates within a strain are sequenced.

Overall, our new reference gene set provides a solid foundation for systematically dissecting the function of SARS-CoV-2 proteins, and focusing experimental work on high-confidence uncharacterized ORFs, which can be guided in part by their evolutionary dynamics (such as the rapid evolution of part of ORF6, indicating a possible adaptive role, and the contribution of ORF8 to fitness within an individual but not to transmission). In addition, our gene-level, codon-level, and nucleotide-level *Sarbecovirus* constraint, and the classification of all existing and potential SNVs and known RNA modification sites into likely-functional vs. likely-neutral based on their evolutionary history, provide important foundations for elucidating SARS-CoV-2 biology, understanding its evolutionary dynamics, prioritizing candidate driver mutations among co-inherited mutations, and prioritizing candidate regions for vaccine design and refinement.

## Methods

### Genomes and alignments

Genome sequences were obtained from https://www.ncbi.nlm.nih.gov/. The genomes and NCBI annotations for SARS-CoV-2 and SARS-CoV were obtained from the records for accessions NC_045512.2 and NC_004718.3, respectively. The UniProt annotations for SARS-CoV-2 were obtained from the UCSC Genome Browser^42^ on April 5, 2020. Note that UniProt later updated their annotations, based in part on the preprint of this manuscript.

The 44 *Sarbecovirus* genomes used in this study were selected starting from all *Betacoronavirus* and unclassified coronavirus full genomes listed on ncbi via searches https://www.ncbi.nlm.nih.gov/nuccore/?term=txid694002[Organism:exp] and the same with txid1986197 and txid2664420 on 5-Mar-2020, excluding any that differed from NC_045512.2 in more than 10,000 positions in a pairwise alignment computed using the Apr-02-2012 version of NW-align^92^ (obtained from https://zhanglab.ccmb.med.umich.edu/NW-align/), that cutoff being chosen so as to distinguish *Sarbecovirus* genomes among those that were classified, and removing near duplicates, including all SARS-CoV and SARS-CoV-2 genomes other than the reference. Coronavirus genomes in the left half of Fig. 3 were those listed by https://www.ncbi.nlm.nih.gov/genomes/GenomesGroup.cgi?taxid=11118 on February 11, 2020.

The genomes were aligned using clustalo^93^ with the default parameters. The phylogenetic tree was calculated using RAxML^94^ using the GTRCATX model. Clustalo version 1.2.3 was obtained from http://www.clustal.org/omega/clustal-omega-1.2.3-macosx. RAxML was obtained from https://github.com/stamatak/standard-RAxML.git on Sep-22-2020, commit a33ff40640b4a76abd5ea3a9e2f57b7dd8d854f6 Tuesday May 29 06:28:07 2018+0200.

### PhyloCSF, FRESCo, and other conservation metrics

PhyloCSF (Phylogenetic Codon Substitution Frequencies)^32^ determines whether a given nucleotide sequence is likely to represent a functional, conserved protein-coding sequence by determining the likelihood ratio of its multi-species alignment under protein-coding and non-coding models of evolution that use pre-computed substitution frequencies for every possible pair of codons, and codon frequencies for every codon, trained on whole-genome data. PhyloCSF software was obtained from git@github.com:mlin/PhyloCSF.git on Aug-28-2014, commit e8378dadc3d0fe039828530c53b5e6787f8bf682 Thu Aug 28 15:34:58 2014-0400. PhyloCSF was run using the 29mammals empirical codon matrices but with the *Sarbecovirus* tree substituted for the mammals tree. Input alignments were extracted from the whole-genome alignment and columns containing a gap in the reference sequence were removed. Browser tracks were created as was done previously for other species^34^: PhyloCSF was run using -strategy=fixed on every codon in each frame and scores were smoothed using an HMM having four states, one representing coding regions and three representing noncoding regions, with the emission of each codon being its PhyloCSF score. Scores listed in Supplementary Data 2 were calculated on the local alignment for each ORF or mature protein, excluding the final stop codon, using the default PhyloCSF parameters, including -strategy=mle.

FRESCo software was obtained from the supplementary data in the publication that introduced FRESCo^39^ and was run using HYPHY version 2.220180618beta(MP) for Linux on x86_64 on 9-codon windows in each of the NCBI annotated ORFs. Alignments were extracted for the ORF excluding the final stop codon, and gaps in the reference sequence were removed. SCEs were found by taking all windows having synonymous rate less than 1 and nominal *P*-value < 10^−5^, and combining overlapping or adjacent windows. For the mutation analysis, FRESCo was also run on 1-codon windows using codon alignments (Supplementary Data 14) constructed as follows: amino acid sequences for each gene were aligned; excessively divergent, long, or short genes were removed; and the amino acid alignment was used as a guide to construct a codon alignment.

Substitutions per site and per neutral site for each annotated ORF and mature protein were calculated by extracting the alignment column for each site or, respectively, 4-fold degenerate site, from the whole-genome alignment and determining the parsimonious number of substitutions using the whole-genome phylogenetic tree. For columns in which some genomes did not have an aligned nucleotide, the number of substitutions was scaled up by the branch length of the entire tree divided by the branch length of the tree of genomes having an aligned nucleotide in that column.

PhastCons and phyloP tracks shown in Fig. 2 are the Comparative Genomics tracks from the UCSC Genome Browser, which were constructed (by UCSC) from a multiz^95^ alignment of the list of 44 *Sarbecovirus* genomes that we supplied to UCSC. PhastCons and phyloP scores were downloaded from the UCSC Table Browser (group: Comparative Genomics; track: 44 Bat CoVs; table: Bat PhyloP or Bat PhastCons (strainPhyloP44way)) on 2021-01-10, and averaged over each ORF and mature protein to obtain the scores in Supplementary Data 2.

### Variant analysis

Single nucleotide variants were downloaded from the “Nextstrain Vars” track in the UCSC Table Browser on 2020-04-18 at 11:46 AM EDT. Table S3 includes one additional mutation, G24047A, from a later download, in order to represent Korber substitution A829T/S. We defined an amino acid to be “conserved” if there were no amino-acid-changing substitutions in the *Sarbecovirus* alignment of its codon. We defined codons to be “synonymously constrained” if the synonymous rate at that codon calculated by FRESCo using 1-codon windows was <1.0 with nominal *P*-value < 0.034, corresponding to a false discovery rate of 0.125. We defined an intergenic nucleotide to be “conserved” if there were no substitutions of that nucleotide in the *Sarbecovirus* alignment. We classified SNVs as Synonymous, Nonsynonymous, or Noncoding, relative to the NCBI annotations, so SNVs within ORF10 were classified as coding, and SNVs within overlapping ORFs 3c and 9b were classified relative to the longer containing ORFs 3a and N, respectively. However, in Supplementary Data 3, we also classified mutations according to our proposed reference gene annotations (fields beginning with New_); when classifying mutations in overlapping ORFs 3a/3c and N/9b we classify SNVs relative to the ORF in which the mutation is non-synonymous if that is true for only one of the frames, or the ORF for which the amino acid change is more radical (as defined by the blosum62 matrix obtained from biopython version 1.58^96^) if it is non-synonymous in both frames, or the larger ORF if the mutation is synonymous in both frames.

We determined mature proteins for which the density of amino-acid-changing SNVs differed significantly from the density that would be expected from their level of conservation, by calculating the residual of a linear regression of amino-acid-changing SNV density as a function of the fraction of conserved amino acids, for all mature proteins. The regression line was *y* = 0.235-0.165*x*. We determined significance using a binomial *p*-value with a false discovery rate cutoff of 0.05. To further test significance of the SNV depletion in S1, we downloaded a larger set of SNVs from the UCSC Table Browser as above on 2020-05-09.

The 16 spike-protein substitutions prioritized were those reported by Korber et al. in their bioRxiv preprint^63^ or later *Cell* publication^64^ (ones at >0.3% frequency, or 0.1% if near certain epitopes). The mutations defining the other lineages were those reported by Rambaut et al. ^70^ for B.1.1.7, by Tegally et al. ^75^ for B.1.351, and by Naveca et al. ^78^ for P.1 and P.2.

To find regions that were significantly enriched for missense mutations in conserved amino acids, we first defined a null model as follows. For each mature protein, we counted the number of missense mutations and the number of conserved amino acids and randomly assigned each SNV to a conserved amino acid in the same mature protein (using Python’s random.randint function), allowing multiplicity. For any positive integer *n*, we found the largest number of mutations that had been assigned to any set of *n* consecutive conserved amino acids within the same mature protein across the whole genome. Doing this 100,000 times gave us a distribution of the number of missense mutations in the most enriched set of *n* consecutive conserved amino acids in the genome. Comparing the number of actual missense mutations in any particular set of *n* consecutive conserved amino acids to this distribution gave us a nominal *p*-value for that *n*. We applied this procedure for each *n* from 1 to 100 and multiplied the resulting *p*-values by a Bonferroni correction of 100 to calculate a corrected *p*-value for a particular region to be significantly enriched. We note that these 100 hypotheses are correlated because enriched regions of different lengths can overlap, so a Bonferroni correction is overly conservative and our reported *p*-value of 0.012 understates the level of statistical significance. To find significantly depleted regions we applied a similar procedure with every *n* from 1 to 1000, but did not find any depleted regions with nominal *P*-value <0.05 even without multiple hypothesis correction.

B.1.1.7 isolates having mutations near the ORF8-truncating mutation Q27* (C27972T) were found by downloading the GISAID database^97^ 2021-02-05_08-24.fasta.gz from https://www.gisaid.org; restricting to sequences with pangolin_lineage B.1.1.7; excluding sequences with bases other than A, C, G, or T; and finding sequences that do not contain the 15-nt context TGTACTTAACATCAA around the C27972T mutation. The 14 sequences in which the nonsense mutation had reverted to the reference sense codon are Belgium/rega-an374/2020, Belgium/rega-an375/2020, Belgium/rega-an376/2020, England/QEUH-109B25C/2021, France/HDF-IPP01172/2021, India/GJ-GBRC-452/2020, Ireland/D-NVRL-20IRL12095/2020, Netherlands/NB-RIVM-10628/2021, Netherlands/ZE-RIVM-10631/2021, Netherlands/ZH-RIVM-10634/2021, Spain/CT-HUVH-76625/2021, Switzerland/un-UHB-30830994/2020, UnitedArabEmirates/4362/2020, and USA/DE-DHSS-FLW00689808A/2021. The six others with nearby mutations are England/210291775/2021, England/ALDP-FB6B45/2021, Wales/ALDP-FB5074/2021, England/ALDP-1013483/2021, England/ALDP-10EC896/2021, and England/MILK-112DC7E/2021. We found that there were at least seven independent reversion events by classifying the genomes containing the reversions into distinct lineages using the branch-defining mutations in the Nextstrain^98^ tree for 20I/501Y.V1 updated 2021-02-08 showing 175 of 3863 genomes sampled between December 2020 and January 2021.

### Miscellaneous

Ribosome footprints shown in Fig. 5 are from the track hub at ftp://ftp-igor.weizmann.ac.il/pub/hubSARSRibo.txt20 accessed on 2020-05-30.

Statistics on short viral proteins were calculated by counting all proteins having protein-level evidence and not flagged as “Fragment” in the list of viral protein sequences in the manually curated UniProtKB/Swiss-Prot database^99^, release 2020_06, release date 02-Dec-2020, downloaded from ftp://ftp.uniprot.org/pub/databases/uniprot/current_release/knowledgebase/taxonomic_divisions/uniprot_sprot_viruses.dat.gz on 2021-01-09.

Statistics were calculated using R version 3.4.4, Python 2.7, or Excel for Mac 2011. Statistical significance was defined as *P* < 0.05.

### Data access

The PhyloCSF tracks and FRESCo SCEs are available for the SARS-CoV-2/wuhCor1 assembly in the UCSC Genome Browser at http://genome.ucsc.edu as public track hubs^1,37,38,42^ named “PhyloCSF” and “Synonymous Constraint”. The alignments and phylogenetic tree used here are provided as supplementary materials (Supplementary Data 12 and 13, respectively). The alignments may be viewed, color coded to indicate protein-coding signatures, using CodAlignView (https://data.broadinstitute.org/compbio1/cav.php) with alignment set wuhCor1_c and chromosome name NC_045512v2. The FRESCo output files for 9- and 1-codon windows are provided as supplementary materials (Supplementary Data 7 and  8, respectively).

Our proposed reference gene set for SARS-CoV-2 and the set of candidate genes that we have rejected are included in BED format in supplementary materials (Supplementary Data 10 and  11, respectively) and are available as the “PhyloCSF Genes” track in the UCSC Genome Browser (the track showing the candidate genes we have rejected may be displayed using the configuration page).

A browser track showing SARS-CoV-2 single nucleotide variants, color coded by whether they are non-coding, synonymous, or amino-acid-changing, and whether they are in conserved codons, as well as tracks showing all codons that are conserved at the amino acid or synonymous level, may be viewed in the UCSC Genome Browser using the track hub at https://data.broadinstitute.org/compbio1/SARS-CoV-2conservation/trackHub/hub.txt. The details page for each SNV includes information about *Sarbecovirus* conservation and a link to view the alignment of a neighborhood of the SNV in CodAlignView.

In this resource, we have augmented mutation data made available by UCSC^100^ with our own annotations. UCSC data came from nextstrain.org^98^, which was derived from genome sequences deposited in GISAID (https://www.gisaid.org)^97^. Right of use and publication of the underlying sequences is entirely controlled by the authors of the original resource and the contributors of individual sequences, who are acknowledged in the Nextstrain metadata file (Supplementary Data 16) Our analysis provides an additional layer of annotation on their work rather than replicating or replacing it.

Original data usage policy as provided by UCSC: “The data presented here is intended to rapidly disseminate analysis of important pathogens. Unpublished data is included with permission of the data generators and does not impact their right to publish. Please contact the respective authors (available via the Nextstrain metadata.tsv file) if you intend to carry out further research using their data. Derived data, such as phylogenies, can be downloaded from nextstrain.org (see “DOWNLOAD DATA” link at bottom of page)—please contact the relevant authors where appropriate.”

### Reporting summary

Further information on research design is available in the Nature Research Reporting Summary linked to this article.

## Supplementary information

Supplementary Information

Reporting Summary

Description of Additional Supplementary Files

Supplementary Data 1

Supplementary Data 2

Supplementary Data 3

Supplementary Data 4

Supplementary Data 5

Supplementary Data 6

Supplementary Data 7

Supplementary Data 8

Supplementary Data 9

Supplementary Data 10

Supplementary Data 11

Supplementary Data 12

Supplementary Data 13

Supplementary Data 14

Supplementary Data 15

Supplementary Data 16

## Data Availability

The PhyloCSF tracks and FRESCo synonymous constraint elements are available for the SARS-CoV-2/wuhCor1 assembly in the UCSC Genome Browser at http://genome.ucsc.edu as public track hubs^1,37,38,42^ named “PhyloCSF” and “Synonymous Constraint”. All other data generated or analysed during this study are included in this published article and its supplementary information files. This study made use of publicly available datasets from GISAID (https://www.gisaid.org) and from UniProtKB/Swiss-Prot (https://www.uniprot.org). Source data are provided with this paper.

## Source data

Source Data

## Code availability

All of our computer code and algorithms used during this study were previously published and are publicly available, namely PhyloCSF^32^, FRESCo^39^, and algorithms to create PhyloCSF browser tracks^34^.
