# Supplementary material for: SARS-CoV-2 gene content and COVID-19 mutation impact by comparing 44 Sarbecovirus genomes
Source: Nat Commun. 2021 May 11;12:2642. doi: 10.1038/s41467-021-22905-7 (PMC8113528; doi:10.1038/s41467-021-22905-7)
Supplement: Supplementary file 1 — Supplementary Information [file 41467_2021_22905_MOESM1_ESM.pdf]

SARS-CoV-2 gene content and COVID-19 mutation impact by comparing 44 *Sarbecovirus* genomes

Supplementary Figures 2
- **Supplementary Figure 1. PhyloCSF signal for polyprotein.** 2
- **Supplementary Figure 2. Cross-strain nucleotide identity.** 2
- **Supplementary Figure 3. Branch-length-adjusted PhyloCSF score strongly rejects ORF10.** 3
- **Supplementary Figure 4. Alignment of nsp11 and frameshift site** 4
- **Supplementary Figure 5. ORF8 Phylogeny.** 5
- **Supplementary Figure 6. Nucleocapsid-overlapping ORF9c in both reading frames.** 6
- **Supplementary Figure 7. Nucleocapsid-overlapping ORF9b in both reading frames.** 7
- **Supplementary Figure 8. Alignments of rejected ORFs** 8
- **Supplementary Figure 9. ORF3b alignment in two frames.** 9
- **Supplementary Figure 10. ORF2b** 10
- **Supplementary Figure 11. SNV-depleted regions.** 11
- **Supplementary Figure 12. Single nucleotide variants and conservation.** 12
- **Supplementary Figure 13. Pangolin comparison of ORF3d.** 13
- **Supplementary Figure 14. Substitutions in rapidly spreading lineages.** 14

Supplementary Notes 15
- **Supplementary Note 1 Resolution of ambiguous gene names** 15
- **Supplementary Note 2 Process for distinguishing protein-coding ORFs** 15
  - Conserved non-overlapping ORFs 15
  - Conserved overlapping ORFs 15
  - Lineage or strain-specific ORFs 16
- **Supplementary Note 3 Search for other novel protein-coding genes** 16
- **Supplementary Note 4 Evaluating evidence regarding functional translation of ORF3d and ORF3d-2** 17
- **Supplementary Note 5 Experimental evidence supporting each ORF** 18
- **Supplementary Note 6 Effect of reference gene set on mutation classification** 19
- **Supplementary Note 7 Possible explanations for within-strain/across-strains deviation in nsp3 and S1** 19
- **Supplementary Note 8 Enriched and depleted clusters of missense SNVs** 19

Supplementary References 20

Supplementary Figures

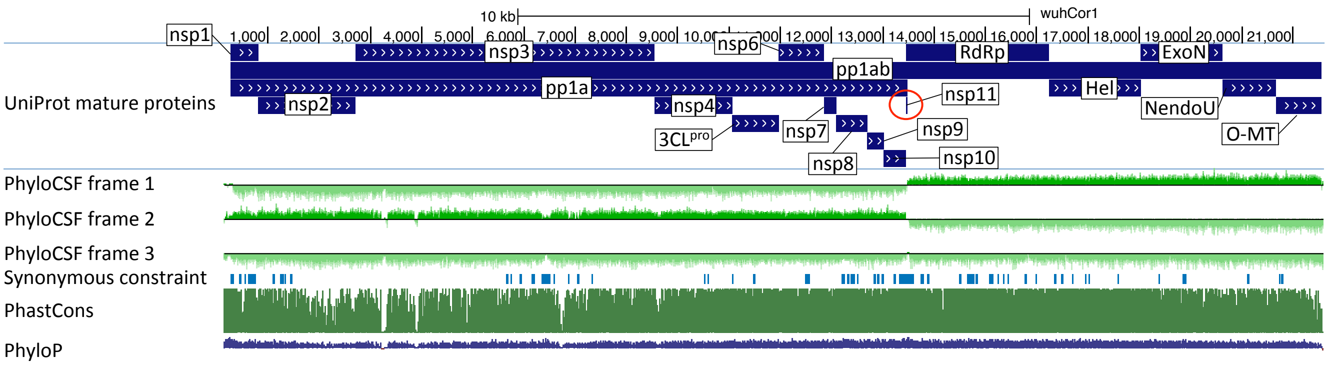

Supplementary Figure 1. PhyloCSF signal for polyprotein.

UCSC Genome Browser view SARS-CoV-2 genome for ORF1a and ORF1b encoding polyprotein pp1a and jointly via frameshift pp1ab, showing gene annotations for individual non-structural proteins (nsp), PhyloCSF tracks (green) in each of 3 reading frames, and Synonymous Constraint Elements (SCEs, blue), along with phastCons/phyloP nucleotide-level constraint (green/blue). Polyprotein pp1a is processed into mature peptides nsp1-nsp11 and pp1ab into peptides nsp1-10 and nsp12-16. PhyloCSF signal shows clear protein-coding signal for all proteins, as expected for functional proteins (except nsp11, red circle, discussed in the main text). PhyloCSF signal captures the correct frame throughout the entire length of each protein (except nsp3, where some small regions show reduced frame-2 signal and/or increased frame-3 signal, but upon inspection these are stop-codon-free only in frame-2 and do not represent dual-coding candidates).

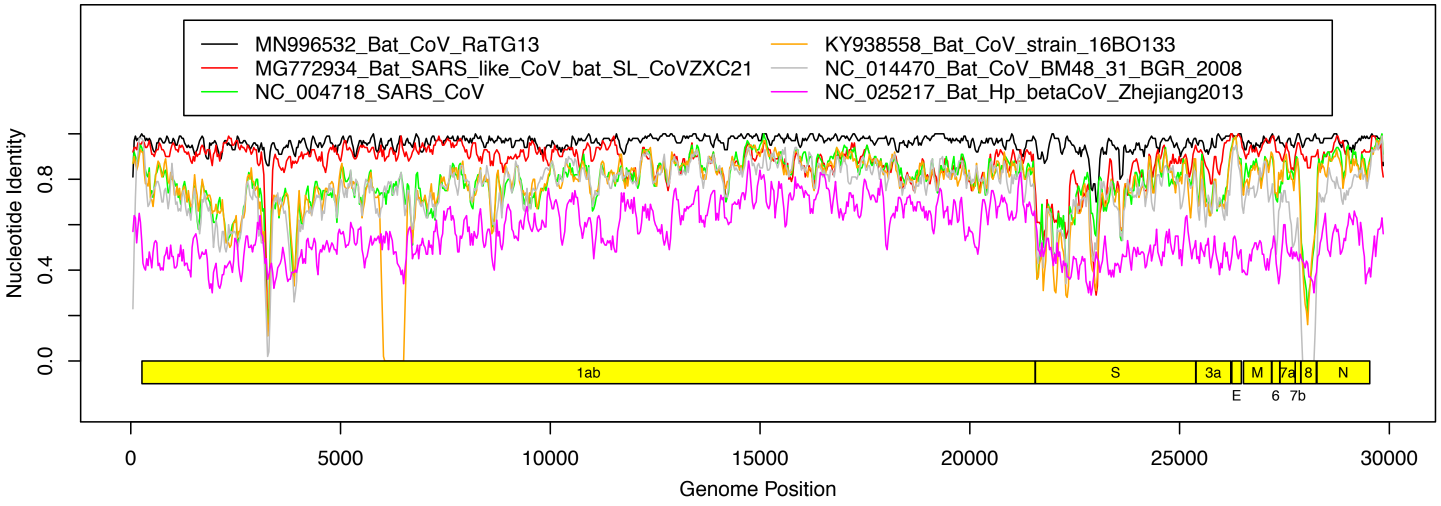

Supplementary Figure 2. Cross-strain nucleotide identity.

Fraction of nucleotides that are identical between SARS-CoV-2 and various other strains in 100 nt windows covering the genome, with 30 nt overlap between neighboring windows. Strains shown are the two closest sarbecoviruses to SARS-CoV-2, namely RaTG13 and ZXC21; the farthest *Sarbecovirus* strain BM48_31_BGR_2008; SARS-CoV; the strain farthest from SARS-CoV within its subclade, 16BO133; and the closest outgroup strain to the sarbecoviruses, namely the *Hibecovirus* Bat_Hp_betaCoV_Zhejiang2013. Regions where the order of strains changes, such as ORF8 and the 5' end of S, suggest evolutionary histories that include recombination events.

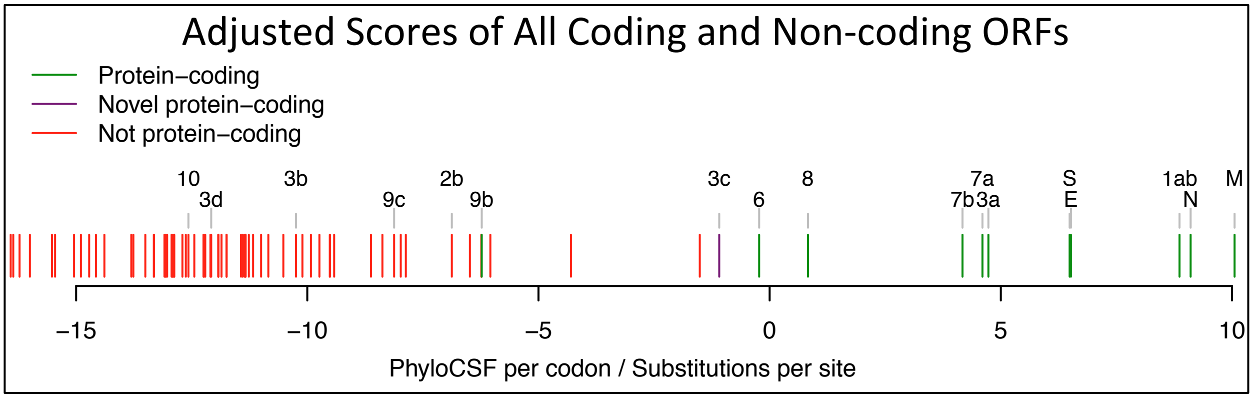

Supplementary Figure 3. Branch-length-adjusted PhyloCSF score strongly rejects ORF10.
Similar to **Fig. 1c**, but showing PhyloCSF scores per codon divided by the average number of substitutions per site, to adjust for the fact that high-nucleotide-conservation regions show compressed unscaled PhyloCSF scores (closer to zero) because there are fewer nucleotide substitution events. The branch-length-scaled score distribution further separates the scores of confirmed protein-coding genes (green) from non-protein-coding segments (red). The very low score of ORF10 with this adjustment indicates that its only-slightly-negative unscaled-PhyloCSF score in **Fig. 1c** stems from the high nucleotide conservation of the region, rather than protein-coding constraint. The scores of N-overlapping ORFs 9b and 9c are both reduced, consistent with the high nucleotide conservation of N. Notably, the branch-length-adjusted score for 3c remains high, consistent with its protein-coding nature, and despite the higher overall nucleotide conservation of its dual-coding region. We have manually inspected all other candidates with adjusted scores higher than 9c, and all are rejected as not protein-coding: two are discussed in **Supplementary Fig. 8**, and the remaining all show internal stop codons.

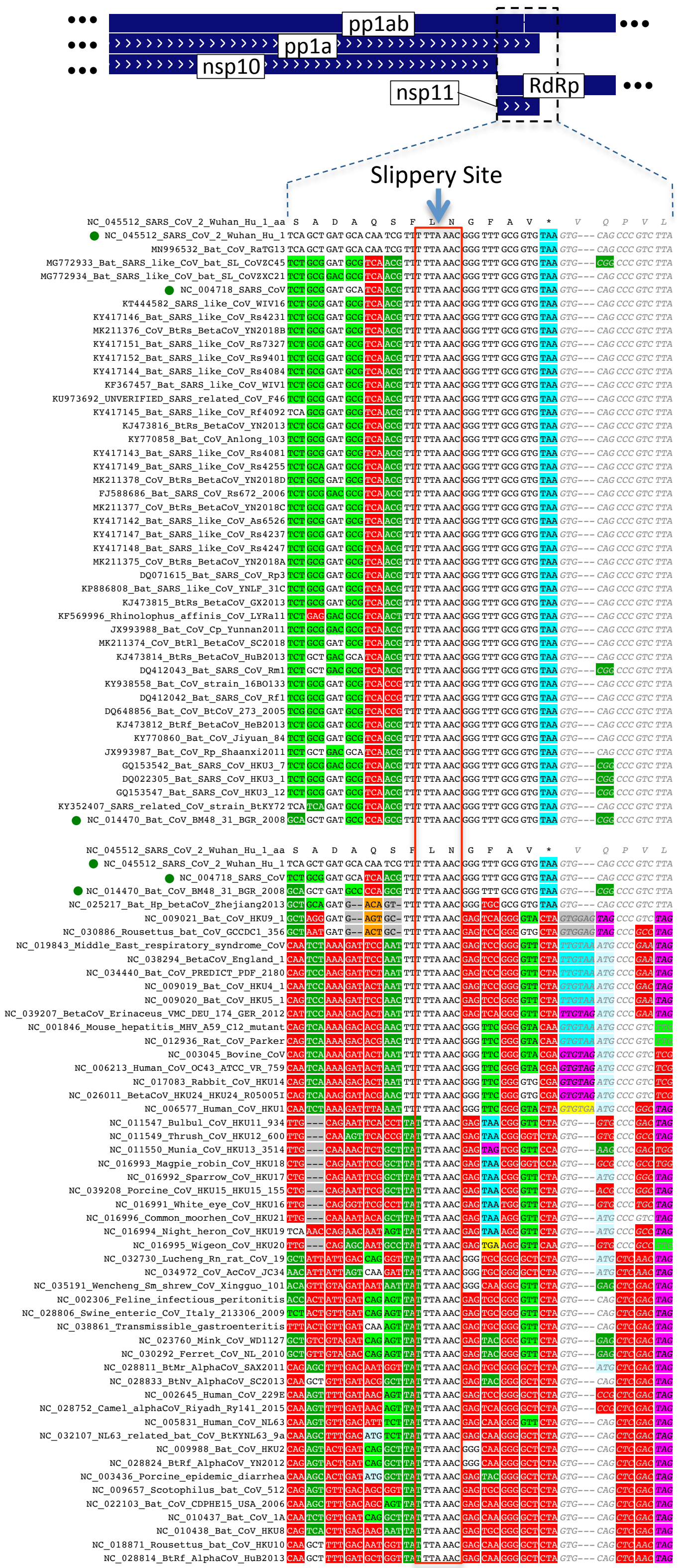

Supplementary Figure 4. Alignment of nsp11 and frameshift site.

Alignment of genomic region encoding nonstructural protein nsp11 and subsequent 5 codons in 44 sarbecoviruses (top) and 52 coronavirinae (bottom, green dots for strains included in both). Programmed frameshift slippery site (red rectangle) is perfectly conserved in all genomes. Genomic region encoding RdRp shares the 5' nine codons with nsp11 but then translation continues 3' of the slippery site in a different reading frame. The four codons 3' of the slippery site are perfectly conserved among known sarbecoviruses, consistent with a dual coding region, but nsp11 is only 13 amino acids long and its stop codon is poorly conserved in coronavirinae (cyan, magenta, and yellow stop codons), suggesting that it is not a functional protein.

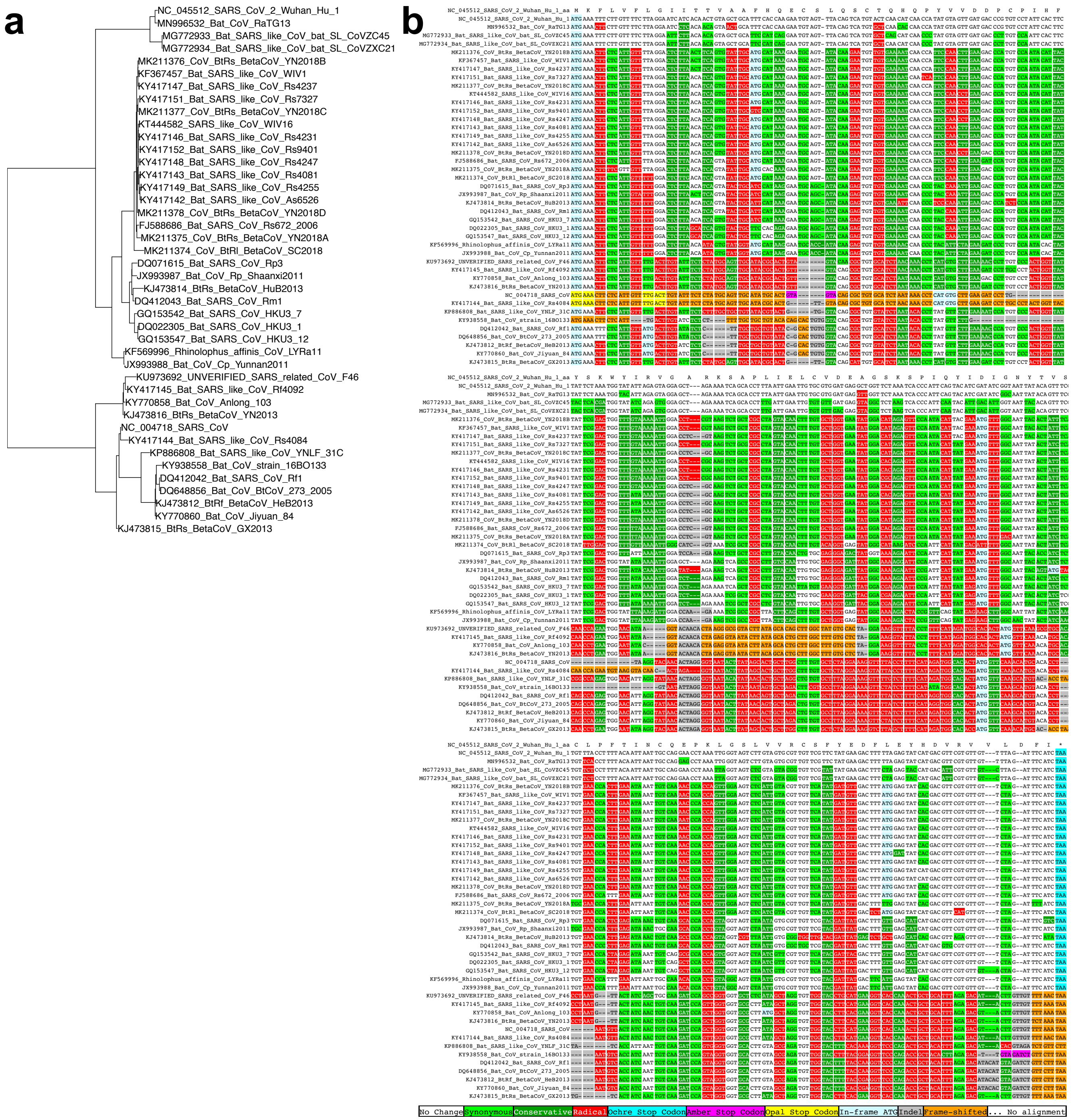

Supplementary Figure 5. ORF8 Phylogeny.

a. Phylogenetic tree computed from ORF8 alignment differs from genome-wide tree in **Fig. 3**, indicating there has been recombination. **b.** Alignment of ORF8 with strains ordered according to the ORF8 tree shows radical amino acid substitutions (red) disrupting almost every residue. Although the apparent high rate of nucleotide-level evolution in ORF8 when estimated using the whole genome tree (3.9 substitutions per site, 6.2 per four-fold degenerate site) is partly an artifact of estimation along a tree topology that does not match the history of ORF8, it remains very high (2.1 and 3.7 respectively, **Supplementary Data 2**) when estimated using the ORF8-specific phylogeny, substantially higher than other ORFs (e.g. 1.1 and 2.8 respectively for ORF1ab when restricting to the 42 strains aligned in ORF8), and thus is not entirely an artifact of recombination.

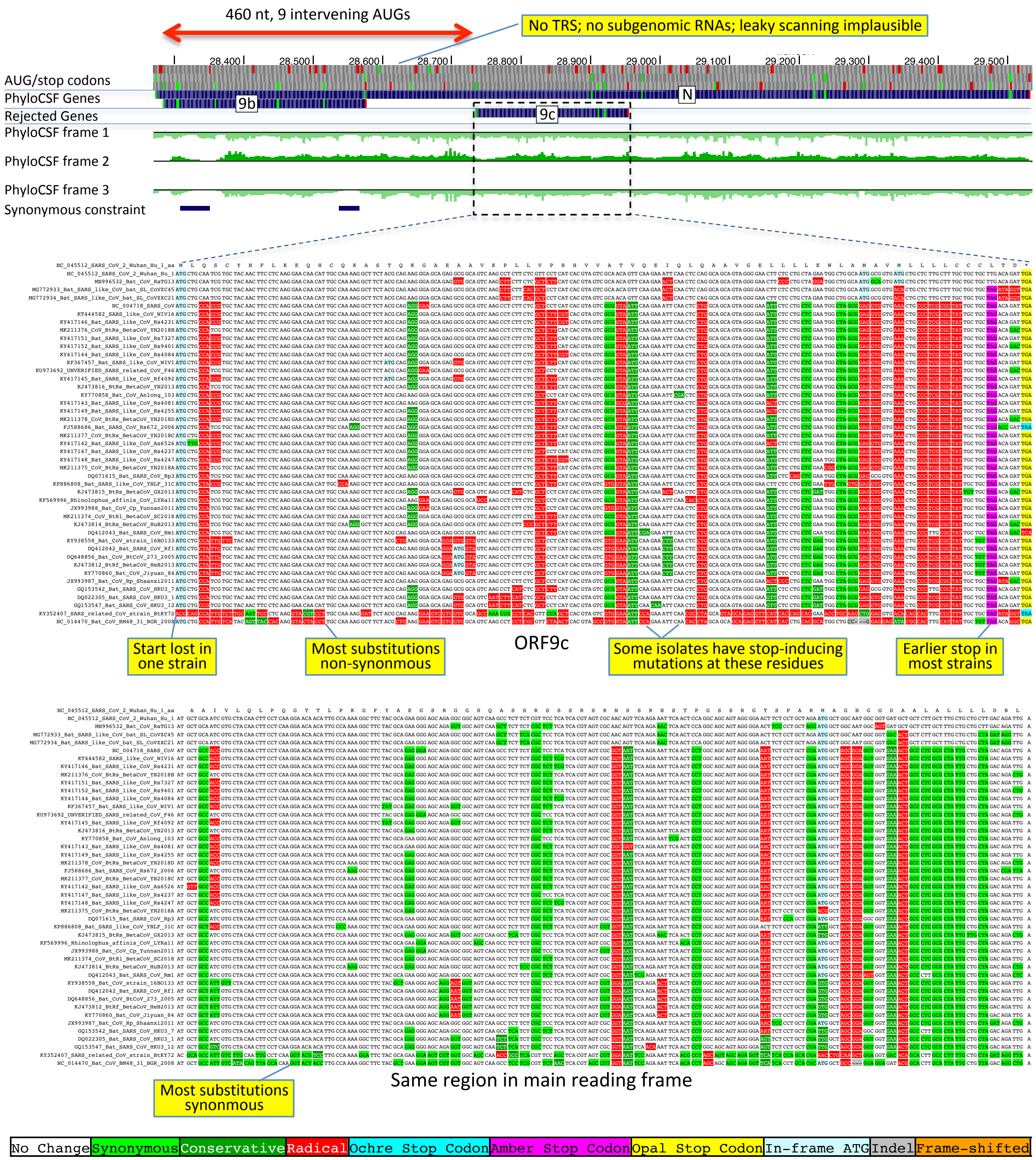

Supplementary Figure 6. Nucleocapsid-overlapping ORF9c in both reading frames.
ORF9c in the genomic context of the nucleocapsid gene (N) (top), and *Sarbecovirus* alignment of this region in the reading frames of ORF9c (middle) and the nucleocapsid gene (bottom). The start codon of ORF9c is 460 nucleotides after N's with 9 intervening AUG codons making it unlikely that ORF9c translated via leaky ribosomal scanning from the subgenomic RNA for N. Other evidence confirms that ORF9c is not a functional protein-coding gene (**Figure 6**).

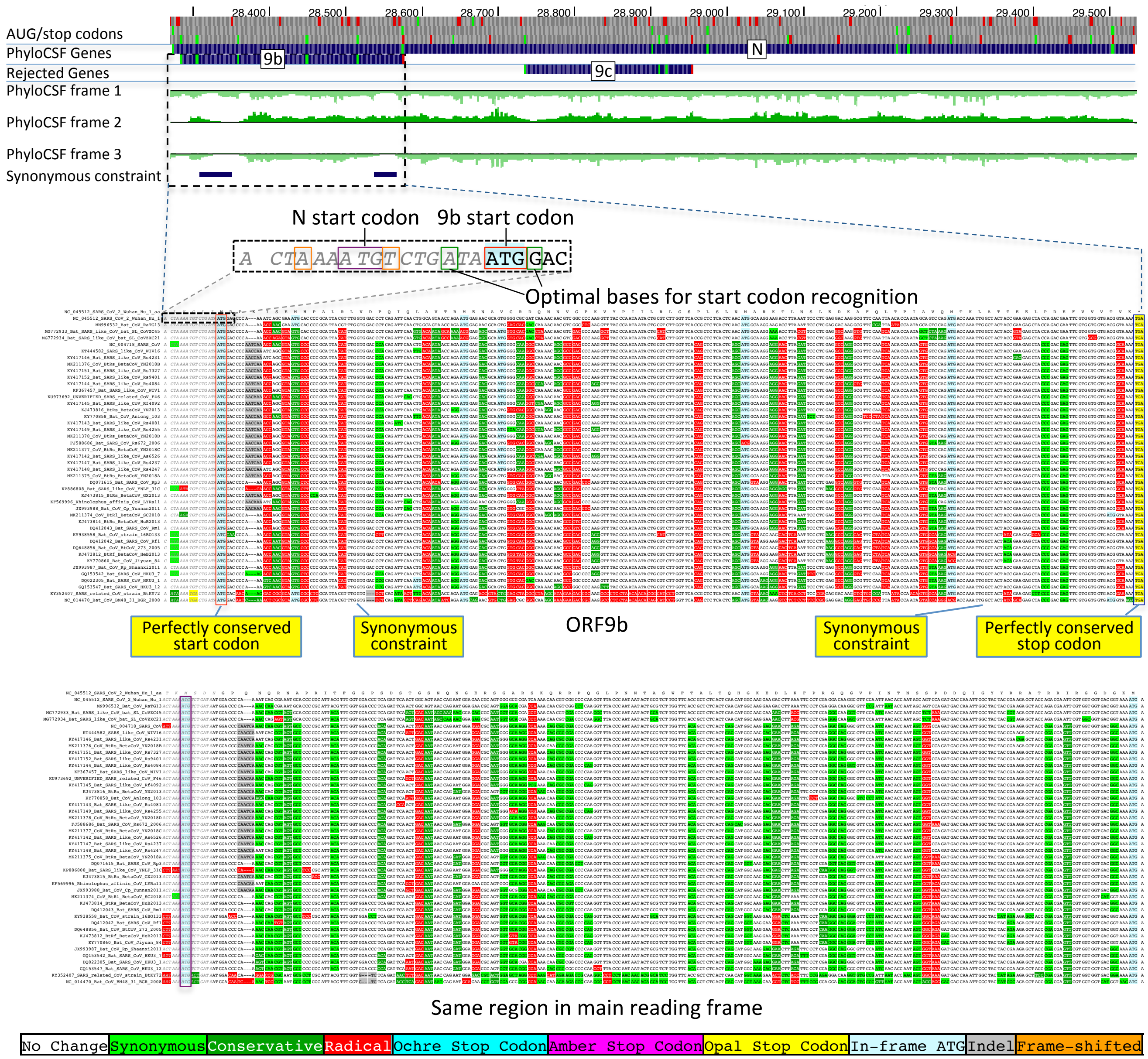

Supplementary Figure 7. Nucleocapsid-overlapping ORF9b in both reading frames.

ORF9b in the genomic context of the nucleocapsid gene (top), and *Sarbecovirus* alignment of this region in the reading frames of ORF9b (middle) and the nucleocapsid gene (bottom).

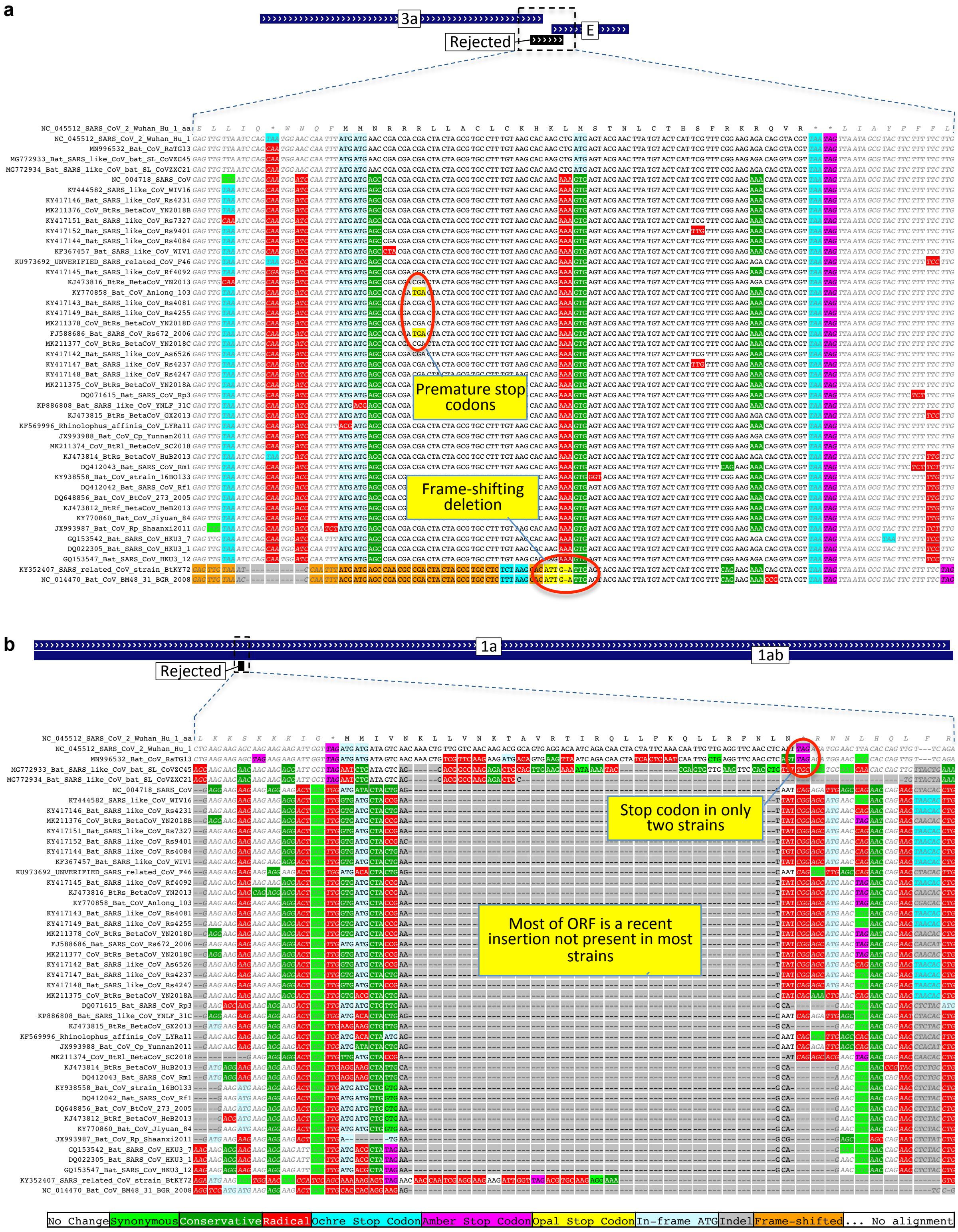

Supplementary Figure 8. Alignments of rejected ORFs.

CodAlignView images of ORFs rejected during our search for novel conserved coding regions. **a.** 32-codon ORF (26183-26278) that overlaps the 3' end of ORF3a and the 5' end of E with PhyloCSF score -2.74. Two strains have a frame-shifting one-base deletion within the ORF, and two others have premature stop codons. None of the substitutions are synonymous. There is high nucleotide-level constraint, but it continues on both sides of the ORF, suggesting it does not result from translation of the ORF. This ORF is the 3' end of the region orthologous to SARS-CoV ORF3b (which is interrupted by multiple stop codons in SARS-CoV-2). **b.** 31-codon ORF (3207-3299) overlapping ORF1a, with PhyloCSF score -7.77. Most of the ORF consists of a 75-nt insertion that is only present in SARS-CoV-2, RaTG13, and CoVZC45, and the start and stop codons are missing in CoVZC45, so this is not a conserved coding sequence.

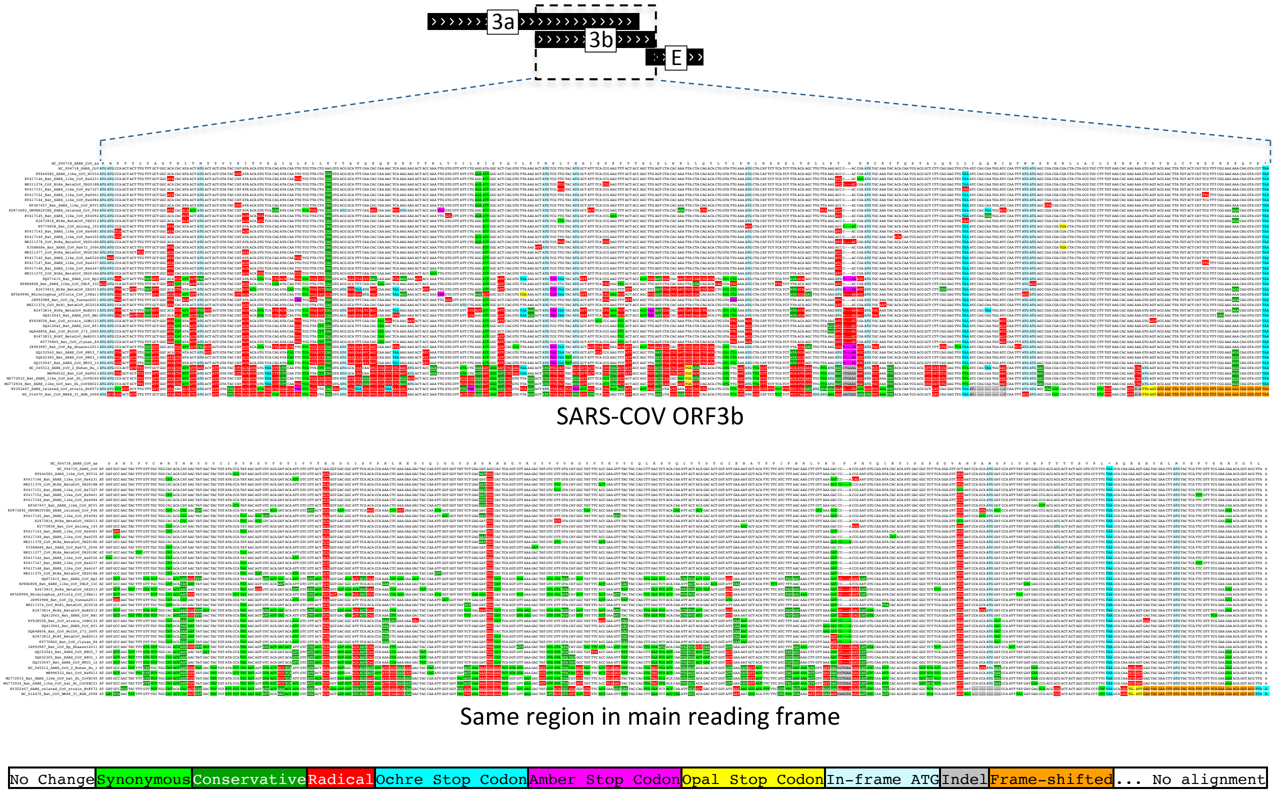

Supplementary Figure 9. ORF3b alignment in two frames.
Alignment of SARS-CoV ORF3b (as in **Fig. 8** but without wrapping), and the same region in the reading frame of ORF3a.

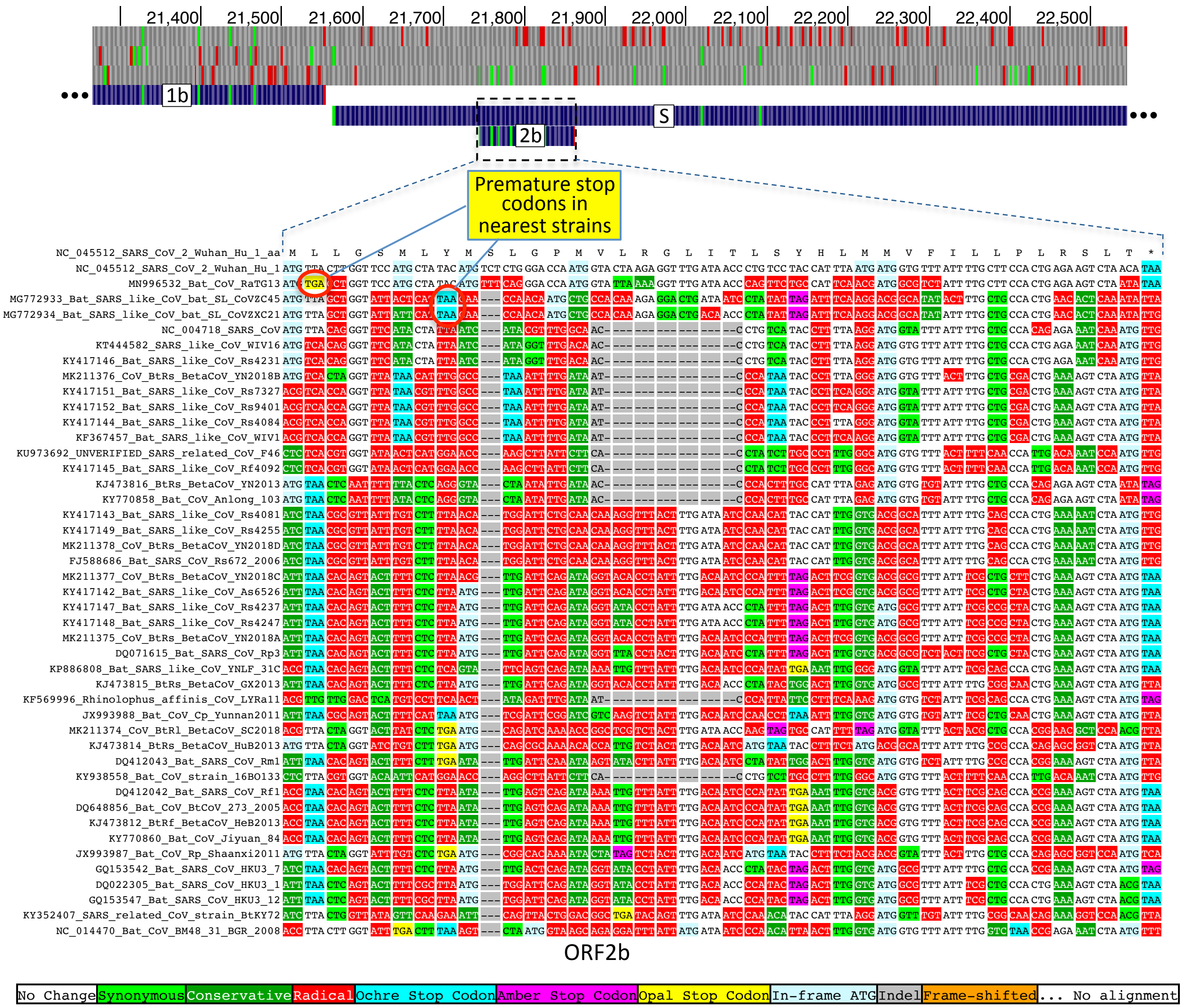

Supplementary Figure 10. ORF2b.

Alignment of *Sarbecovirus* genomes at 39-codon ORF2b overlapping S near its 5' end. Premature stop codons (yellow, cyan, and magenta) truncate the ORF in most strains including the three closest to SARS-CoV-2, and the start and stop codon of ORF2b are poorly conserved, indicating that this ORF is not a conserved protein-coding gene. Although translation of this ORF in SARS-CoV-2 was predicted based on ribosome profiling data, presumably through leaky scanning of the S subgenomic RNA, there is no evidence that the resulting short peptide is stable and contributes to viral fitness.

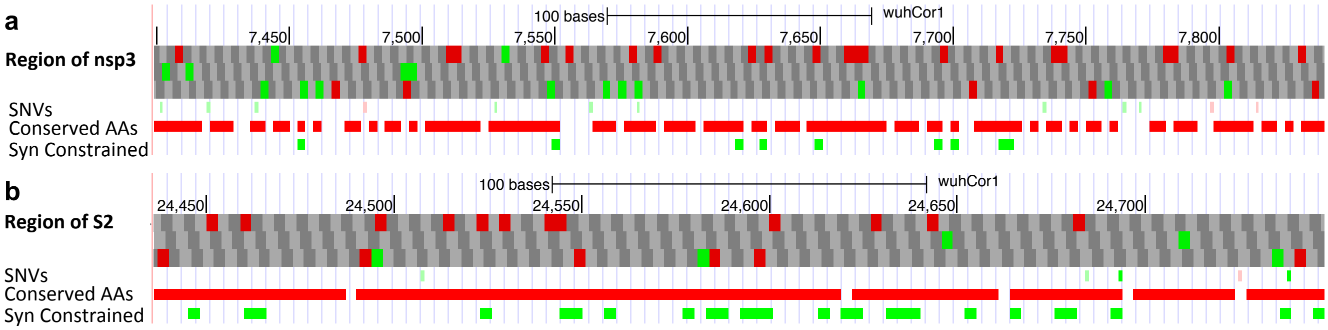

Supplementary Figure 11. SNV-depleted regions.

UCSC Genome Browser images of regions in nsp3 (**a**) and S2 (**b**). Most amino acids in these regions are conserved (red rectangles in Conserved AAs track), but the only missense mutations (light red rectangles in SNVs track) disrupt non-conserved amino acids (mutations disrupting conserved amino acids would be bright red if present). The lack of missense mutations in such a large set of conserved amino acid residues could indicate that constraint in the *Sarbecovirus* clade has continued particularly strongly in the SARS-CoV-2 population. However, although these are the most depleted regions in the genome for missense mutations in conserved amino acid residues, neither depletion is statistically significant, even without any correction for multiple region lengths searched (nominal p=0.072 and p=0.093, respectively).

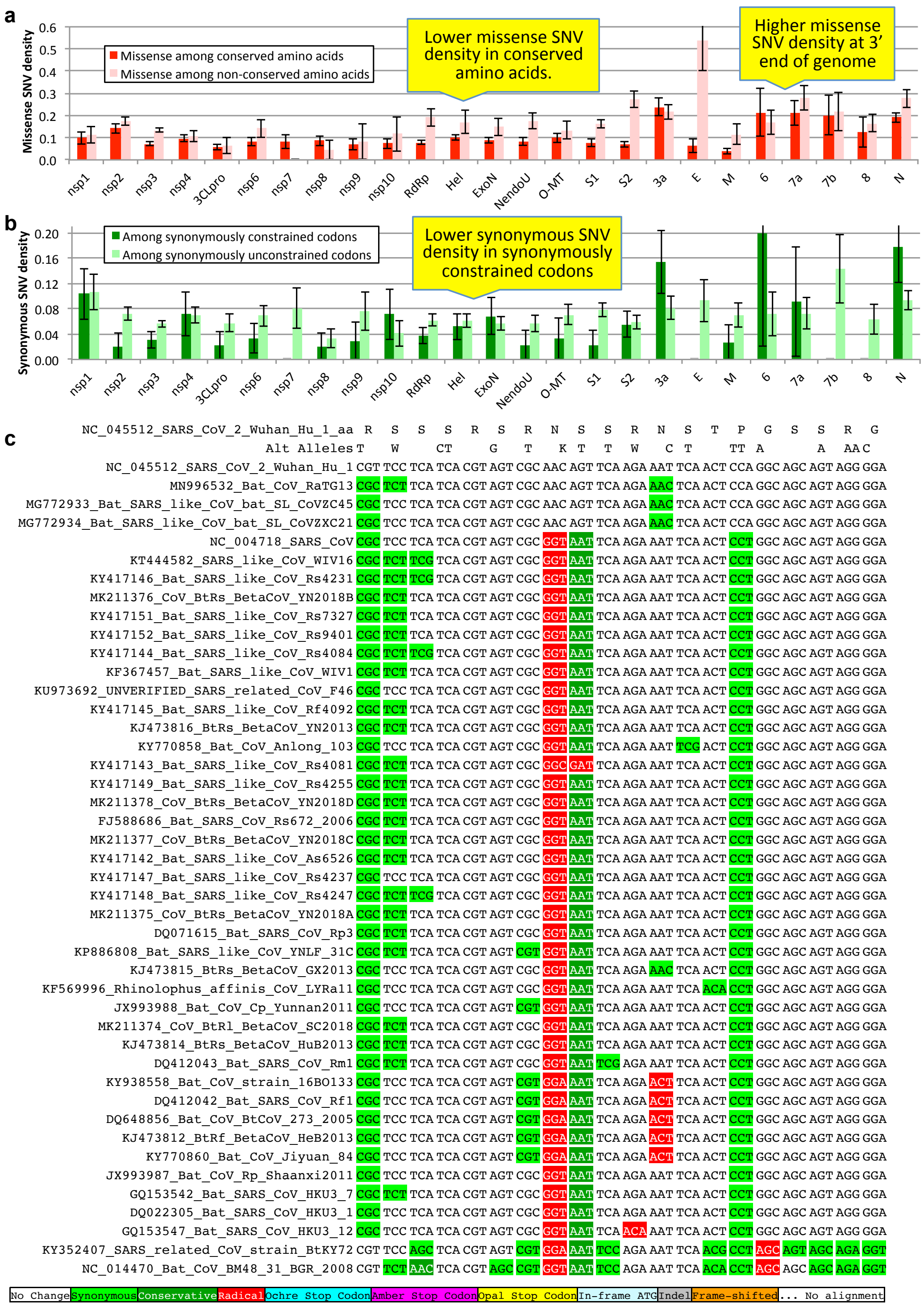

Supplementary Figure 12. Single nucleotide variants and conservation.

Error bars indicate standard error of mean. **a.** Density of SNVs disrupting conserved amino acids (dark red) is significantly lower than disrupting non-conserved amino acids (light red). Both densities are higher near the 3' end of the genome, indicating higher mutation rate or less purifying selection even among amino acids that are perfectly conserved among known sarbecoviruses. **b.** Density of synonymous mutations in synonymously constrained codons (dark green) is significantly lower than among synonymously unconstrained codons (light green), a depletion seen in most genes. Overall, conservation in the *Sarbecovirus* clade at both the amino acid level and nucleotide level is associated with purifying selection on mutations in the SARS-CoV-2 population. **c.** Alignment of 20 amino acid nucleocapsid-gene region that is highly enriched for mutations disrupting perfectly conserved amino acids (alternate alleles shown in second row, W = A or T, K = G or T). There are 14 non-synonymous mutations among the 14 perfectly conserved amino acids (columns with no red or dark green). This region is contained within a predicted B Cell epitope, suggesting positive selection for immune system avoidance.

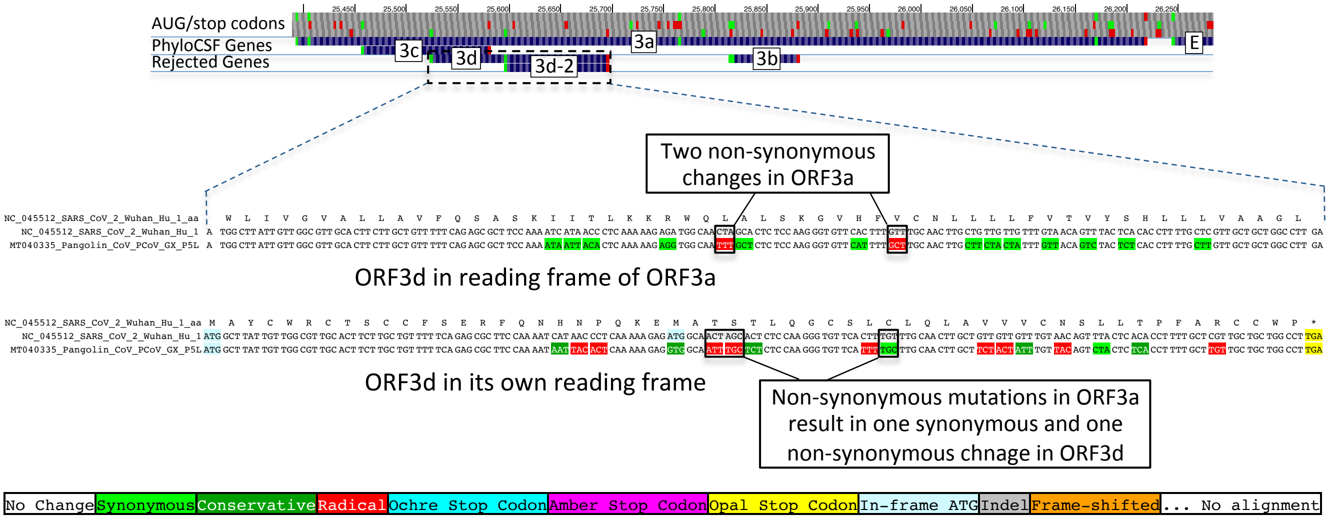

Supplementary Figure 13. Pangolin comparison of ORF3d.

Alignment of SARS-CoV-2 ORF3d to pangolin coronavirus GX/P5L in reading frames of ORF3a (top) and ORF3d (bottom). There are two amino acid differences in the ORF3a reading frame, resulting from CUA-UUU and GUU-GCU codon changes. Nelson et al[1] used OLGenie, a variant of the dN/dS test intended for overlapping reading frames that restricts to sites that are non-synonymous in the main frame, to compare ORF3d in these two strains and reported that the differences were suggestive of constraint. They report that there are 0 or 1 mutations that are non-synonymous in both reading frames and 2 or 1, respectively, that are non-synonymous in the ORF3a frame and synonymous in the ORF3d frame, depending on the order of the two mutations that resulted in the CUA-UUU substitution (assuming all mutations are single nucleotide changes), for an average of 0.5 non-synonymous and 1.5 synonymous changes in the ORF3d frame that are non-synonymous in the ORF3a frame. However, we find that either order of the CUA-UUU change results in one synonymous and one non-synonymous change in the ORF3a frame (CUA[L] -> UUA[L] ->UUU[F], CUA[L] -> CUU[L] ->UUU[F]), both of which are non-synonymous in the ORF3d frame (ACU[T] -> AUU[I] and AGC[S] -> UGC[C]). Combined with the non-synonymous GUU[V]->GCU[A] change in the ORF3a frame that is synonymous in the ORF3d frame (UGU[C] -> UGC[C]), we find that with either order of mutations, two non-synonymous mutations in the ORF3a frame result in 1 synonymous and 1 non-synonymous change in the ORF3d frame. This is not a significant enrichment of synonymous changes and does not suggest constraint.

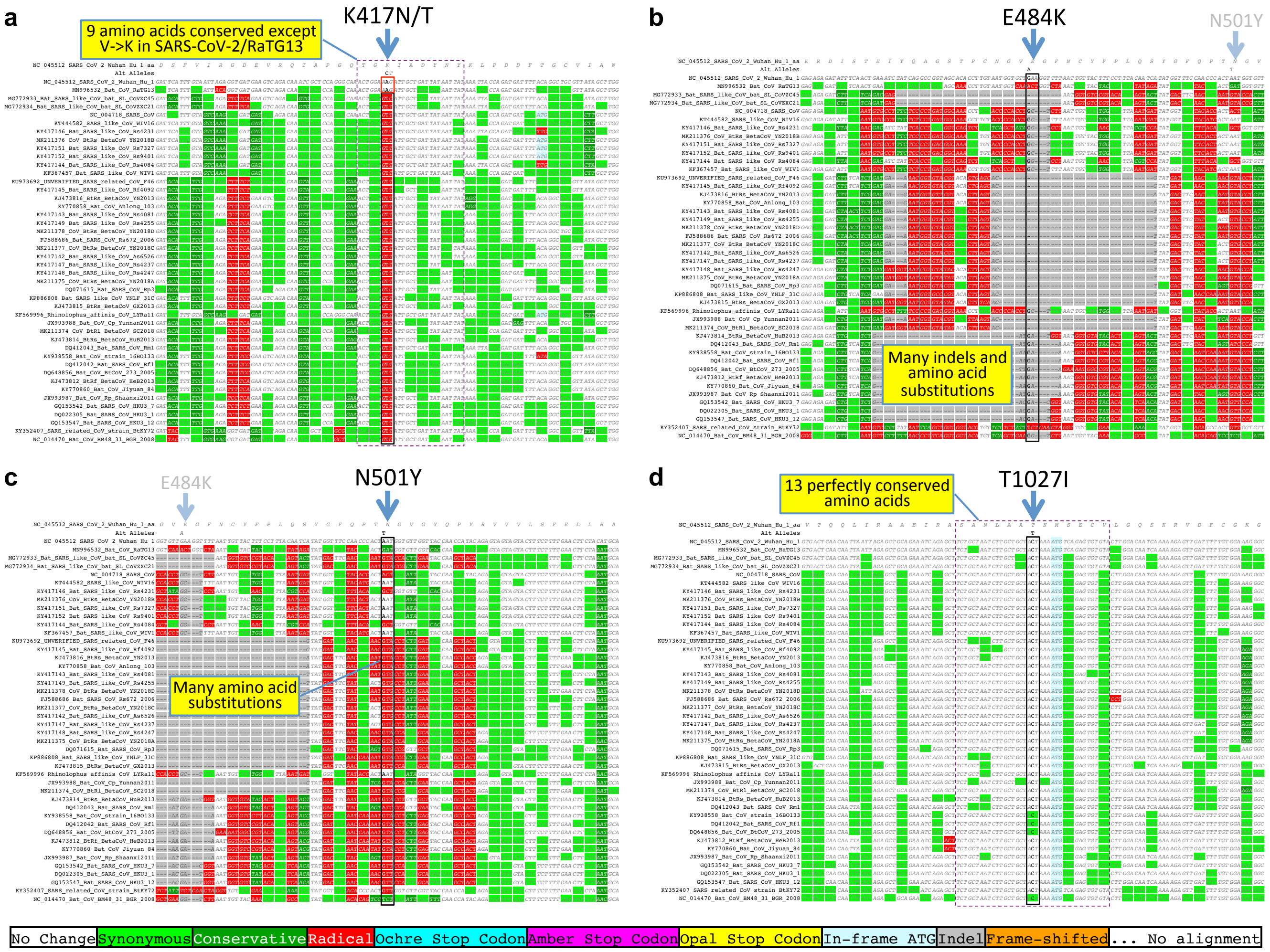

Supplementary Figure 14. Substitutions in rapidly spreading lineages.

CodAlignView alignments of three spike-protein amino acid substitutions in the Receptor Binding Domain (RBD), K417N/T **(a)**, E484K **(b)**, and N501Y**(c)**, that are present in more than one SARS-CoV-2 lineages associated with faster spread or immune system evasion, and one other spike-protein substitution, T1027I **(d)**, that appears in one of these lineages, with 19 codons of context on each side. **(a)** K417N (K417T in P.1), thought to contribute to evasion of monoclonal antibodies and serum antibodies of convalescent and vaccinated patients, is in a string of 9 amino acids that were perfectly conserved among sarbecoviruses (purple box, all codons white indicating no change or light green indicating synonymous) except in the SARS-CoV-2/RaTG13 lineage in which the ancestral valine codon (black box, red GTC and GTT codons) changed to lysine (red box, white AAG codons in SARS-CoV-2/RaTG13). The high conservation in the other 42 strains suggests this residue is functional, but might have changed to a non-optimal amino acid in SARS-CoV-2/RaTG13, perhaps due to drift, in which case this residue would be less constrained in SARS-CoV-2 and more likely to vary in order to escape antibodies generated against the wild type virus. **(b)** E484K (black box), one of the residues in contact with the human ACE2 receptor and also thought to contribute to evasion of wild type antibodies, is in a highly variable region containing many indels (gray dashes) and radical amino acid substitutions (red). The high *Sarbecovirus* variability is consistent with previous evidence of positive selection in SARS-CoV-2 based on an excess of non-synonymous mutations and increasing frequency, and indicates that its rapid evolution is a conserved feature across both small-scale and large-scale evolutionary time. **(c)** N501Y (black box), also in contact with the human ACE2 receptor and thought to contribute to increased transmissibility, is also in a highly variable region, containing many radical (red) and conservative (dark green) amino acid substitutions, once more consistent with previous evidence of positive selection in SARS-CoV-2. **(d)** T1027I (black box) is a radical amino acid change in a string of 13 perfectly conserved amino acid residues (purple box) in a highly conserved region of the S2 spike-protein subunit. It is one of several non-RBD amino acid substitutions in the lineages associated with faster spread or immune system evasion that disrupt conserved residues and are thus likely to have biologically-relevant consequences.

Supplementary Notes

Supplementary Note 1 Resolution of ambiguous gene names

The ORFs overlapping S, ORF3a, and N have been referred to by different names by different authors, and different ORFs have been referred to by the same name. We have resolved these inconsistently used names in communication with several other authors and in consultation with members of the ICTV Coronavirus Study Group[2]. The ORFs, in 5' to 3' order, with our name, length and coordinates (not including the stop codon), and other names that have been used are:

Resolution of ambiguous gene names.

Our name	Length (codons)	Coordinates	Other names/Notes
ORF2b	39	21744-21860	S.iORF1
ORF3c	41	25457-25579	ORF3h, 3a.iORF1, ORF3b
ORF3d	57	25524-25694	ORF3b
ORF3d-2	33	25596-25694	3a.iORF2
ORF3b	22	25814-25879	5’ end of SARS-CoV ORF3b ortholog
ORF9b	97	28284-28574	ORF9a, N.iORF1
ORF9c	73	28734-28952	ORF9b, ORF14

Supplementary Note 2 Process for distinguishing protein-coding ORFs

Here we describe our process, summarized in **Fig. 4,** for distinguishing protein-coding ORFs, which we have previously applied to ORFs on transcripts in several eukaryotic species. We distinguish the cases of conserved non-overlapping ORFs, conserved overlapping ORFs, and lineage or strain-specific ORFs. One condition that applies to all three is that ORFs more than a few hundred codons long are almost certainly protein coding because the probability that a nucleotide sequence that long would contain no in-frame stop codons would be vanishingly small unless there were selective pressure to prevent them from being introduced. While a precise cutoff will depend on the desired confidence level and other properties of the sequence, we note that the negative strand of the SARS-CoV-2 genome includes stop to stop sequences up to 159 codons long, and found that among 1000 random sequences of 30,000 nucleotides the longest stop to stop sequence was 289 codons long, so 300 codons might be a reasonable threshold above which any ORF is almost certainly protein-coding but below which more evidence is required. Conversely, very short ORFs are rarely protein-coding -- among the 3054 manually curated full-length viral proteins with protein-level evidence in the UniProtKB/Swiss-Prot database, 99% are 56 amino acids or longer, and only seven are less than 40 amino acids long.

Conserved non-overlapping ORFs

By definition, for an ORF to encode a *conserved* functional protein there must exist homologous ORFs of comparable length in all or most strains of the clade. In that case, a high PhyloCSF score for the entire ORF provides evidence of protein-coding function since any score greater than 0 implies the alignment is more likely under our coding model of evolution than our non-coding model. However, some rapidly evolving or alternate-frame ORFs do not have positive PhyloCSF score over the full length of the ORF even if they encode functional proteins. In some cases a substantial subset of an ORF will have a high PhyloCSF score, which provides evidence that the subset is protein-coding; this in turn implies that the whole ORF is protein-coding unless there is some alternative means by which that subset could be translated, such as initiation at a downstream start codon, or, in the case of eukaryotic genomes, alternative splicing.

Conserved overlapping ORFs

Evolutionary evidence for protein-coding function of overlapping ORFs is more difficult to resolve, as protein-

coding signatures in the primary reading frame heavily influence scores in alternate frames: they skew the signal as protein-preserving mutations in one frame are typically protein-disruptive in the other, and they compress the signal as there are fewer substitutions. However, conservation of the alternate-frame amino acid sequence leads to a depletion of synonymous substitutions in the primary ORF localized over the overlapping segment, resulting in a strong signal of overlapping-constraint[3–5]. While synonymous constraint extending over all or most of an ORF is a strong indication of coding in an alternate frame, some overlapping ORFs exhibit synonymous constraint over only part of the ORF, with the remaining portions rapidly evolving in the alternate frame. A caveat is that synonymous constraint can also result from other kinds of overlapping elements, though generally these will be shorter than a complete ORF. Consequently, we would prefer to see experimental evidence of translation to boost confidence that the synonymous constraint signal is truly due to coding in two reading frames.

While PhyloCSF scores for protein-coding overlapping ORFs are depressed by the constraint on the main ORF, they will still typically be higher than scores of non-coding overlapping ORFs, so this can be used as an additional indication of protein-coding evolution.

Although ideally the start and stop codon of a conserved ORF would themselves be conserved, we do not consider extending or shortening of the ORF at either end by a few codons to be disqualifying. In contrast, we consider in-frame stop codons or frameshifting indels in some strains that are far from either end of the ORF as stronger evidence against protein-coding status. If a protein *does* remain functional in a strain even though it is substantially lengthened or shortened relative to other strains, we would expect the portion that is common to the homologs in all or most strains, and thus has been evolving as a protein-coding region, to have a high PhyloCSF score, or a high degree of synonymous constraint in the case of overlapping ORFs.

Lineage or strain-specific ORFs

Finally, we consider ORFs that have arisen *de novo* in some lineage or even a single strain. If the lineage includes sufficient evolutionary distance then the above-described methods may be used, but if the ORF is present in only a single strain or a set of very closely related strains then methods other than cross-strain comparative information must be used. In principle, the ratio of non-synonymous to synonymous SNVs can give information about protein-coding constraint, but in our experience this information is rarely statistically significant for short ORFs and especially for short overlapping ORFs. Allele frequencies could theoretically provide more information, but are confounded by hitchhiking, founder effects, and heterogeneous environmental constraints.

Consequently, we are left with experimental information such as ribosome profiling to demonstrate that an ORF is translated, keeping in mind that even if it is translated, additional experimental information is needed to demonstrate that the protein is functional, i.e., contributes to viral fitness.

Although we have not found SNVs to be useful for demonstrating that an ORF is protein-coding, SNVs that introduce a stop codon or frameshifting insertions or deletions can provide an indication that an ORF is *not* protein-coding. Although mutations that disable a functional but inessential ORF might exist at low levels in a population, if the ORF encodes a protein that contributes to viral fitness then the decreased fitness resulting from its loss will eventually make itself felt and prevent the mutation from remaining at high frequency in the population.

Supplementary Note 3 Search for other novel protein-coding genes

As discussed in the main text, we computed PhyloCSF scores for all 67 non-NCBI-annotated AUG-to-stop locally-maximal SARS-CoV-2 ORFs ≥25 codons long and found that the top scoring candidate, ORF3c, is protein-coding.

Continuing down the sorted list in order of decreasing PhyloCSF score per codon, we found the candidate ORF with the next best score (-2.74) is a 32-codon ORF (26183-26278) that overlaps the 3' end of ORF3a and the 5' end of E (**Supplementary Fig. 8a**) and is orthologous to the 5' end of SARS-CoV ORF3b. Two strains have a frame-shifting 1-base deletion within the ORF, and two others have premature stop codons. None of

the substitutions are synonymous. There is high nucleotide-level constraint, but it continues on both sides of the ORF, suggesting it results from something other than translation of the ORF. Overall, this ORF does not show the evolutionary signature of a functional coding sequence. Next in the list is ORF9b which we have discussed elsewhere. Fourth is a 31-codon ORF (3207-3299) overlapping ORF1a, having PhyloCSF score -7.77 (**Supplementary Fig. 8b**). Most of this ORF consists of a 75-nt insertion that is only present in SARS-CoV-2, RaTG13, and CoVZC45, and the start and stop codons are missing in CoVZC45, so this is not a conserved coding sequence. Finally, the fifth-ranked candidate is ORF9c, which we have discussed elsewhere.

The relatively high scores of ORFs 9b and 9c among these 67 ORFs are, in part, an artifact of the low density of substitutions throughout N, which they both overlap. This low density, which is found even in the parts of N that are not in ORFs 9b or 9c, decreases the number of substitutions available to PhyloCSF for distinguishing its coding and noncoding evolutionary models, which compresses the PhyloCSF score towards 0, resulting in a better rank among the negative scores. If we compensate for this by dividing by the average number of substitutions per site in the ORF, ORFs 9b and 9c, while still in the top half, move down to the 92nd and 83rd percentile among the 67 ORFs considered, whereas ORF3c remains the best scoring-candidate (**Supplementary Fig. 3**).

To search for additional novel protein-coding regions, we relaxed our criteria to include ORFs having at least 10 codons, allow near-cognate start codons, and allow ORFs contained within another ORF in the same frame. The most promising were two non-canonical ORFs in the 5'-UTR with slightly positive PhyloCSF score, 14-codon CUG-initiated NC_045512.2:92-133 and 31-codon AGG initiated NC_045512.2:158-250. However, neither are among the translated ORF candidates predicted by ribosome profiling[6], and the evolutionary evidence is not strong enough to consider it likely that such short non-canonical ORFs generate proteins. Because it has been conjectured that translation might occur on the negative-strand genomic and subgenomic RNAs that are intermediates in viral gene expression and replication in positive-strand RNA viruses [7,8], we also scored ORFs on the negative strand, but again found no convincing candidates. **Supplementary Data 4** contains the complete list of ORFs, with scores and other pertinent information.

Supplementary Note 4 Evaluating evidence regarding functional translation of ORF3d and ORF3d-2

Here, we review and evaluate evidence previously presented in support of translation and protein-coding function of ORF3d and ORF3d-2. Both ORF3d and ORF3d-2 have premature stop codons in all of the other strains of our Sarbecovirus alignment, indicating unambiguously that they do not encode *conserved* proteins (**Fig. 9**), but some researchers have suggested that one or both are newly evolved proteins in SARS-CoV-2. Although proteins do arise *de novo* in viruses, absent evolutionary evidence of purifying selection in a large clade, other sources of evidence must exceed a high threshold of confidence to determine that an ORF encodes a functional protein.

Here, we review the previously proposed evidence for functional translation, and conclude that it is not sufficient:

- A ribosome profiling experiment predicted translation for ORF3d-2 but not ORF3d[6].
- Antibodies that react to a peptide translated from the ORF3d sequence were found in serum from former COVID-19 patients[9] suggesting that ORF3d or its shorter isoform, ORF3d-2, is expressed at sufficient levels to generate an antibody response, but this does not distinguish between the two isoforms or provide evidence that the protein is functional in the sense of contributing to viral fitness.
- ORF3d was found to be significantly longer than expected given the sequence of the main ORF, ORF3a, using the Schlub permutation test (p=0.01)[1,10]. However the p-value is not statistically significant after applying a multiple hypothesis correction for the 12 genes tested; the test is confounded by overlap with ORF3c; and ORF3d is not significantly longer than expected (even without multiple hypothesis correction) when compared to the synonymous mutation null model, which is more biologically realistic than the null model used in the permutation test.
- Re-analysis of the ribosome footprints from Finkel et al[6] found a peak in lactimidomycin and harringtonine reads (which concentrate at translation initiation sites) at the start site of ORF3d[1].

However, the peak read count is considerably lower than for known protein-coding genes and it is unclear whether it is biologically relevant. A graph of read counts averaged over 30-nt windows for a subset of reads for which the translational reading frame could be accurately distinguished found a raised plateau of reads in the ORF3d frame near the middle of ORF3d. However, the raw data show that this is an artifact of the 30-nt window averaging spreading a peak in a single codon at the start site of ORF3d-2, and is thus evidence of translation initiation for ORF3d-2 rather than translation elongation for ORF3d.

- Two algorithms predicted no T-cell epitopes in ORF3d-2 (p=0.001)[1]. This suggests ORF3d-2 is translated but does not clearly indicate that it is functional since selection for immune avoidance would favor mutations that remove epitopes from any translation product, functional or not. There was an overall depletion in predicted T-cell epitopes in ORF3d, but this is likely due to the extreme depletion in the ORF3d-2 subset so it does not provide evidence of translation of ORF3d.
- ORF3d is present in pangolin viruses GX/P5L and GX/P4L but not in the more closely related pangolin GD/1 or bat RaTG13, suggesting it evolved independently or was independently lost several times[1]. An investigation of purifying selection on the amino acid sequence of ORF3d by comparing SARS-CoV-2 to pangolin GX/P5L using OLGenie, a variant of the dN/dS test intended for overlapping reading frames that restricts to sites that are non-synonymous in the main frame, did not find significant evidence of purifying selection, as there are only two non-synonymous substitutions in the main frame, one synonymous and one non-synonymous in the alternate frame (**Supplementary Fig. 13**).
- A study of sequence features that distinguish overlapping from non-overlapping viral ORFs found that various sequence features of ORF3d distinguished it from non-overlapping ORFs, but the study provided no indication of whether this result was statistically significant[11].
- ORF3d[12] was found to have interferon antagonist properties when overexpressed from a plasmid, but the study did not provide evidence that it is expressed from viral RNA during the course of infection.
- A nonsense mutation, G25563U, that truncates ORF3d (but not ORF3d-2), has been found in 24% of over 100,000 complete viral genomes analyzed[1,12,13], demonstrating that ORF3d is not essential for viral replication and transmission. This mutation also causes amino acid changes in ORF3a and ORF3c whose effect on the virus is unknown, and its epidemiological trajectory could also be influenced by other mutations in its haplotype or environmental effects, but it seems unlikely that such effects could compensate for the loss of a viral protein and allow the mutation to persist at high frequency if that protein contributes substantially to viral fitness.
- ORF3d-2 is quite short for a functional protein (33 codons); only four of the 3054 viral proteins having protein-level evidence in the UniProtKB/Swiss-Prot database are as short as or shorter than ORF3d-2.

Thus, while multiple lines of evidence have been proposed, none are conclusive and some argue against functional translation of ORF3d and ORF3d-2. For ORF3d-2, we conclude that there is some evidence of translation, but no evidence that it encodes a functional protein that contributes to viral fitness. For ORF3d, its translation level is substantially lower than that of known proteins, and the high prevalence of a loss-of-function mutation indicates it is unlikely to encode a protein that contributes substantially to viral fitness.

Supplementary Note 5 Experimental evidence supporting each ORF

Among previously ambiguous cases, our new reference gene set includes 3c and 9b, and excludes 3d, 3b, 10, and 9c. These inclusion/exclusion decisions are supported by the experimental evidence. Direct RNA sequencing detected little or no subgenomic RNA for the excluded ORFs[14–17], whereas included ORFs 3c and 9b are near the 5' ends of ORFs 3a and N, where they could be translated via leaky scanning from the corresponding subgenomic RNAs. Translation of 3c and 9b, but not 3d, 3b, or 9c, was predicted in ribosome profiling experiments[6], and footprints in ORF10 appear to support non-functional translation of overlapping ORFs instead. Peptides supporting translation of 9b but not 10 or 9c were detected in proteomics experiments [16,18] (2b, 3c, 3d, and 3b were not included in the search space). Finally, there is a wealth of experimental evidence supporting translation of all of the other named and unnamed ORFs[6,14–18]. We have also excluded ORF2b and ORF3d-2, which were predicted to be translated using ribosome profiling data[6] but are short, non-conserved, and have no experimental evidence of encoding functional proteins that contribute to viral fitness. This information is summarized in **Supplementary Data 2**.

Supplementary Note 6 Effect of reference gene set on mutation classification

Having correct gene annotations is critical for determining the effects of mutations because the first step in mutation classification is understanding how each mutation affects protein sequence. Using our reference gene set instead of previous annotations improves the classification of many mutations.

In particular, we found that seven mutations within ORF3c (T25473C, T25476C, G25494T, G25500A, G25500T, C25539T, and C25572T) that were classified by Nextstrain as synonymous based on their predicted effect on ORF3a, but that we now recognize disrupt amino acids in the ORF3c protein, and three mutations (C25493T, G25563T, and A25575C) induce a more radical amino acid change in ORF3c than in ORF3a. Similarly, seven mutations within ORF9b (C28291T, T28297C, C28315T, C28369T, G28378T, C28432T, C28519T) were considered synonymous changes in N according to the NCBI annotations, but disrupt amino acids in ORF9b, and two others (G28300T, G28357T) induce a more radical amino acid change in ORF9b than in N. Nextstrain also classified ten mutations as disrupting amino acids in ORF10 that we now recognize are non-coding mutations, and five mutations as disrupting amino acids in ORF9c that are synonymous in N and therefore do not change any protein.

Supplementary Data 3 includes Nextstrain mutations annotated with respect to the current NCBI reference gene annotations and also with respect to our proposed new reference annotations. The INFO field also includes Nextstrain's classification according to UniProt annotations.

Supplementary Note 7 Possible explanations for within-strain/across-strains deviation in nsp3 and S1

We propose several possible explanations for the depletion of amino-acid-changing mutations in S1 and nsp3 relative to what would be expected from the overall trend given their high inter-strain evolutionary rates. These differences might indicate:

- Recent changes in their mutation rates, or recent changes in their selective pressures, which could be inherent to SARS-CoV-2 independently of the current pandemic, or could stem from different pressures acting on these genes during pandemic expansion in human hosts, relative to stable spread of sarbecoviruses in bat populations
- These differences may also reflect general properties in an adaptation-expansion cycle of coronaviruses, with S1 and nsp3 initially undergoing rapid evolution during adaptation to a new host, followed by a period in which purifying selection suppresses further variation. In this scenario, the smaller-than-expected number of observed mutations in the current pandemic would stem from pre-adaptation of S1 and nsp3, either through transmission in non-human animal hosts with a similar ACE2 receptor, or through undetected transmission in humans prior to when the initial sample for the reference genome was obtained in December 2019 (Wu et al.), thus requiring relatively fewer human-adaptive mutations compared to other genes whose biological functions would adapt to human hosts only later (noting however, that only a subset of mutations in the current pandemic are likely adaptive).
- The frequent recombination observed in S1 suggests an alternative explanation. It is possible that recombination events into our selected 44-strain phylogeny from more distant relatives could have increased the number of inter-strain differences in these genes. In that case, the paucity of mutations in S1 and nsp3 relative to their inter-strain differences might be due to inflation of the latter rather than deflation of the former. However, we note that the amount of distant recombination needed to account for the large discrepancy observed might be implausibly large.
- Lastly, inter-strain differences reflect selective pressures that have acted over evolutionary time scales in which even mildly deleterious mutations are excluded, while within-strain differences reflect smaller evolutionary time scales over which only strongly deleterious mutations are excluded. Thus, the discrepancy observed could also result if the fraction of all possible deleterious amino acid changes of S1 and nsp3 that are strongly deleterious rather than mildly deleterious is sufficiently larger than that fraction for other proteins.

Supplementary Note 8 Enriched and depleted clusters of missense SNVs

Other than the nucleocapsid-gene region that we have already discussed, there are no regions in the genome in which mutations disrupting conserved amino acid residues are significantly denser than would be expected

by chance, given the total number of such mutations in each gene. Nor are there any regions that are significantly depleted for such mutations, which would have indicated regions in which constraint in the *Sarbecovirus* clade has continued particularly strongly in the SARS-CoV-2 population. The regions that are most depleted (though not statistically significantly) have coordinates 7400-7840 in nsp3 with no missense mutations among 103 conserved amino acids and 24437-24748 in S2 with no missense mutations among 99 conserved amino acids (p=0.072 and p=0.093, respectively, without any correction for multiple region lengths searched) (**Supplementary Fig. 11**).

Supplementary References

1. Nelson, C. W. *et al.* Dynamically evolving novel overlapping gene as a factor in the SARS-CoV-2 pandemic. *Elife* **9**, (2020).
2. Jungreis, I. *et al.* Conflicting and ambiguous names of overlapping ORFs in SARS-CoV-2: A homology-based resolution. *Virology* (2021) doi:10.1016/j.virol.2021.02.013.
3. Khan, Y. A. *et al.* Evidence for a novel overlapping coding sequence in POLG initiated at a CUG start codon. *BMC Genet.* **21**, 25 (2020).
4. Sealfon, R. S. *et al.* FRESCo: finding regions of excess synonymous constraint in diverse viruses. *Genome Biol.* **16**, 38 (2015).
5. Lin, M. F. *et al.* Locating protein-coding sequences under selection for additional, overlapping functions in 29 mammalian genomes. *Genome Research* vol. 21 1916–1928 (2011).
6. Finkel, Y. *et al.* The coding capacity of SARS-CoV-2. *Nature* (2020) doi:10.1038/s41586-020-2739-1.
7. Dinan, A. M., Lukhovitskaya, N. I., Olendraite, I. & Firth, A. E. A case for a negative-strand coding sequence in a group of positive-sense RNA viruses. *Virus Evol* **6**, veaa007 (2020).
8. DeRisi, J. L. *et al.* An exploration of ambigrammatic sequences in narnaviruses. *Sci. Rep.* **9**, 17982 (2019).
9. Hachim, A. *et al.* ORF8 and ORF3b antibodies are accurate serological markers of early and late SARS-CoV-2 infection. *Nat. Immunol.* **21**, 1293–1301 (2020).
10. Schlub, T. E., Buchmann, J. P. & Holmes, E. C. A Simple Method to Detect Candidate Overlapping Genes in Viruses Using Single Genome Sequences. *Mol. Biol. Evol.* **35**, 2572–2581 (2018).
11. Pavesi, A. New insights into the evolutionary features of viral overlapping genes by discriminant analysis. *Virology* **546**, 51–66 (2020).
12. Lam, J.-Y. *et al.* Loss of orf3b in the circulating SARS-CoV-2 strains. *Emerg. Microbes Infect.* 1–678

(2020).

13. Gordon, D. E. *et al.* A SARS-CoV-2 protein interaction map reveals targets for drug repurposing. *Nature* (2020) doi:10.1038/s41586-020-2286-9.

14. Kim, D. *et al.* The Architecture of SARS-CoV-2 Transcriptome. *Cell* **181**, 914–921.e10 (2020).

15. Taiaroa, G. *et al.* Direct RNA sequencing and early evolution of SARS-CoV-2. doi:10.1101/2020.03.05.976167.

16. Davidson, A. D. *et al.* Characterisation of the transcriptome and proteome of SARS-CoV-2 reveals a cell passage induced in-frame deletion of the furin-like cleavage site from the spike glycoprotein. *Genome Med.* **12**, 68 (2020).

17. Nomburg, J., Meyerson, M. & DeCaprio, J. A. Pervasive generation of non-canonical subgenomic RNAs by SARS-CoV-2. *Genome Med.* **12**, 108 (2020).

18. Bojkova, D. *et al.* Proteomics of SARS-CoV-2-infected host cells reveals therapy targets. *Nature* **583**, 469–472 (2020).
