# Supplementary material for: SARS-CoV-2 gene content and COVID-19 mutation impact by comparing 44 Sarbecovirus genomes
Source: Nat Commun. 2021 May 11;12:2642. doi: 10.1038/s41467-021-22905-7 (PMC8113528; doi:10.1038/s41467-021-22905-7)
Supplement: Supplementary file 2 — Reporting Summary [file 41467_2021_22905_MOESM2_ESM.pdf]

nature research

Corresponding author(s): Irwin Jungreis and Manolis Kellis

Last updated by author(s): Feb 11, 2021

Reporting Summary

Nature Research wishes to improve the reproducibility of the work that we publish. This form provides structure for consistency and transparency in reporting. For further information on Nature Research policies, see our Editorial Policies and the Editorial Policy Checklist.

Statistics

For all statistical analyses, confirm that the following items are present in the figure legend, table legend, main text, or Methods section.

n/a	Confirmed	
☐	☒	The exact sample size (*n*) for each experimental group/condition, given as a discrete number and unit of measurement
☒	☐	A statement on whether measurements were taken from distinct samples or whether the same sample was measured repeatedly
☐	☒	The statistical test(s) used AND whether they are one- or two-sided<br>*Only common tests should be described solely by name; describe more complex techniques in the Methods section.*
☒	☐	A description of all covariates tested
☐	☒	A description of any assumptions or corrections, such as tests of normality and adjustment for multiple comparisons
☐	☒	A full description of the statistical parameters including central tendency (e.g. means) or other basic estimates (e.g. regression coefficient) AND variation (e.g. standard deviation) or associated estimates of uncertainty (e.g. confidence intervals)
☐	☒	For null hypothesis testing, the test statistic (e.g. *F*, *t*, *r*) with confidence intervals, effect sizes, degrees of freedom and *P* value noted<br>*Give P values as exact values whenever suitable.*
☒	☐	For Bayesian analysis, information on the choice of priors and Markov chain Monte Carlo settings
☒	☐	For hierarchical and complex designs, identification of the appropriate level for tests and full reporting of outcomes
☐	☒	Estimates of effect sizes (e.g. Cohen's *d*, Pearson's *r*), indicating how they were calculated

Our web collection on statistics for biologists contains articles on many of the points above.

Software and code

Policy information about availability of computer code

Data collection	No software was used to collect data. All data used was collected previously by others and obtained by us from publicly available sources.
Data analysis	FRESCo software was obtained from the supplementary data in the publication that introduced FRESCo (Sealfon et al. 2015); there is only one version of FRESCo included in the supplementary data of that publication, it does not get updated, and it has no official version number. FRESCo was run using HYPHY version 2.220180618beta(MP) for Linux on x86_64. PhyloCSF software was obtained from git@github.com:mlin/PhyloCSF.git on Aug-28-2014, commit e8378dadc3d0fe039828530c53b5e6787f8bf682 Thu Aug 28 15:34:58 2014 -0400; there is no version number other than that git commit.. RAxML was obtained from https://github.com/stamatak/standard-RAxML.git on Sep-22-2020, commit a33ff40640b4a76abd5ea3a9e2f57b7dd8d854f6 Tue May 29 06:28:07 2018 +0200; there is no version number other than that git commit. Clustal Omega version 1.2.3 was obtained from http://www.clustal.org/omega/clustal-omega-1.2.3-macosx. The Apr-02-2012 version of NW-align 92 was obtained from https://zhanglab.ccmb.med.umich.edu/NW-align/; there is no version number other than the date. Statistics were calculated using R version 3.4.4, Python 2.7, or Excel for Mac 2011.

For manuscripts utilizing custom algorithms or software that are central to the research but not yet described in published literature, software must be made available to editors and reviewers. We strongly encourage code deposition in a community repository (e.g. GitHub). See the Nature Research guidelines for submitting code & software for further information.

Data

Policy information about availability of data

All manuscripts must include a data availability statement. This statement should provide the following information, where applicable:

- Accession codes, unique identifiers, or web links for publicly available datasets
- A list of figures that have associated raw data
- A description of any restrictions on data availability

The PhyloCSF tracks and FRESCo synonymous constraint elements are available for the SARS-CoV-2/wuhCor1 assembly in the UCSC Genome Browser at http://genome.ucsc.edu as public track hubs (Raney et al. 2014; Wu et al. 2020; Haeussler et al. 2019; Kent et al. 2002) named “PhyloCSF” and “Synonymous Constraint”. All other data generated or analysed during this study are included in this published article and its supplementary information files. Source data for all figures are provided with this paper (Figures 1a, 3a, 10a, and Supplementary Figures 2, 3, and 12). This study made use of publicly available datasets from GISAID (https://www.gisaid.org) and from UniProtKB/Swiss-Prot (https://www.uniprot.org).

Field-specific reporting

Please select the one below that is the best fit for your research. If you are not sure, read the appropriate sections before making your selection.

☐ Life sciences ☐ Behavioural & social sciences ☒ Ecological, evolutionary & environmental sciences

For a reference copy of the document with all sections, see nature.com/documents/nr-reporting-summary-flat.pdf

Ecological, evolutionary & environmental sciences study design

All studies must disclose on these points even when the disclosure is negative.

Study description	Analyzed and compared 44 previously-sequenced Sarbecovirus genomes, and 1875 single-nucleotide variants previously called from 2544 previously sequenced SARS-CoV-2 isolates.
Research sample	Genome sample was 44 previously-sequenced Sarbecovirus genomes, chosen to be at ideal evolutionary distance for detecting protein-coding evolutionary signatures. These were downloaded from NCBI using queries https://www.ncbi.nlm.nih.gov/nuccore/?term=txid694002[Organism:exp] and the same with txid1986197 and txid2664420 on 5-Mar-2020. SNV sample was all variants in the “Nextstrain Vars” track in the UCSC Table Browser (https://www.genome.ucsc.edu/cgi-bin/hgTables/) on 2020-04-18 at 11:46 AM EDT.
Sampling strategy	Our sampling strategy was to exclude any genomes that were incomplete or differed from NC_045512.2 in more than 10,000 positions in a pairwise alignment, that cutoff being chosen so as to distinguish Sarbecovirus genomes among those that were classified, and removing near duplicates, including all SARS-CoV and SARS-CoV-2 genomes other than the reference. For SNVs, we used all available SNVs.
Data collection	Data was collected by downloading genomes from the above mentioned NCBI query and SNVs from the UCSC Table Browser as described above.
Timing and spatial scale	Genomes were those sampled and deposited with NCBI on or before 5-Mar-2020. SNVs were those available in the UCSC Table Browser on 2020-04-18. Both were taken from worldwide samples.
Data exclusions	Genomes were excluded as described in "Sampling strategy" above. No SNVs were excluded.
Reproducibility	All of our analysis should be reproducible using the data described above.
Randomization	When finding regions that were significantly enriched for missense mutations in conserved amino acids, we used Python 2.7's random.randint function.
Blinding	No researchers were blinded.

Did the study involve field work? ☐ Yes ☒ No

Reporting for specific materials, systems and methods

We require information from authors about some types of materials, experimental systems and methods used in many studies. Here, indicate whether each material, system or method listed is relevant to your study. If you are not sure if a list item applies to your research, read the appropriate section before selecting a response.

Materials & experimental systems

n/a	Involved in the study
☒	☐ Antibodies
☒	☐ Eukaryotic cell lines
☒	☐ Palaeontology and archaeology
☒	☐ Animals and other organisms
☒	☐ Human research participants
☒	☐ Clinical data
☒	☐ Dual use research of concern

Methods

n/a	Involved in the study
☒	☐ ChIP-seq
☒	☐ Flow cytometry
☒	☐ MRI-based neuroimaging
