# Supplementary material for: SARS-CoV-2 gene content and COVID-19 mutation impact by comparing 44 Sarbecovirus genomes
Source: Nat Commun. 2021 May 11;12:2642. doi: 10.1038/s41467-021-22905-7 (PMC8113528; doi:10.1038/s41467-021-22905-7)
Supplement: Supplementary file 3 — Description of Additional Supplementary Files [file 41467_2021_22905_MOESM3_ESM.pdf]

Description of Additional Supplementary Files

File Name: Supplementary Data 1
Description: Tab-delimited table with one row for each of 44 *Sarbecovirus* strains used.

File Name: Supplementary Data 2
Description: Tab-delimited table with information on all confirmed or candidate genes and mature proteins of SARS-CoV-2.

File Name: Supplementary Data 3
Description: Tab-delimited table with information about each of the single nucleotide variants used in this study

File Name: Supplementary Data 4
Description: Tab-delimited table listing open reading frames searched for novel coding regions.

File Name: Supplementary Data 5
Description: Tab-delimited table with conservation of RNA modification sites.

File Name: Supplementary Data 6
Description: Tab-delimited table with enrichment analysis for conservation in RNA modification sites.

File Name: Supplementary Data 7
Description: Tab-delimited table with FRESCo output (synonymous and non-synonymous rates) for 9-codon windows.

File Name: Supplementary Data 8
Description: Tab-delimited table with FRESCo output (synonymous and non-synonymous rates) for 1-codon windows.

File Name: Supplementary Data 9
Description: Tab-delimited table with nucleotide identity between SARS-CoV-2 and other strains in 100-nt windows.

File Name: Supplementary Data 10
Description: Our proposed new reference gene set for SARSCoV-2, in BED format.

File Name: Supplementary Data 11
Description: The gene candidates that we rejected, in BED format.

File Name: Supplementary Data 12
Description: Whole-genome alignment of 44 *Sarbecovirus* genomes, in Fasta format.

File Name: Supplementary Data 13
Description: Whole-genome phylogenetic tree, in Newick format.

File Name: Supplementary Data 14
Description: Codon alignments used to run FRESCo for 1-codon windows, in FASTA format.

File Name: Supplementary Data 15
Description: Tab-delimited table with mutations distinguishing B.1.1.7, B.1.351, P.1, and P.2 lineages.

File Name: Supplementary Data 16
Description: List of authors who contributed genomes to GISAID that were used by Nextstrain and UCSC to produce the list of SNVs.
